# α‐1,3‐Glucan Is Required for Cell Wall Integrity and Full Virulence of *Colletotrichum graminicola*, but Not for Evasion of PAMP‐Triggered Immunity

**DOI:** 10.1111/mmi.70114

**Published:** 2026-09-10

**Authors:** Iris Gase, Lala Aliyeva‐Schnorr, Michael Melzer, Maria Ott, Julia Haufe, Matthias Menzel, Timo Engelsdorf, Christiane Rohrbach, Alan de Oliveira Silva, Lars M. Voll, Alexander S. Mickel, Maximilian Groß, Mamoru Wakayama, Holger B. Deising

**Affiliations:** ^1^ Faculty of Natural Sciences III, Institute for Agricultural and Nutritional Sciences, Chair for Phytopathology and Plant Protection Martin‐Luther‐University Halle‐Wittenberg Halle (Saale) Germany; ^2^ Leibniz Institute of Plant Genetics and Crop Plant Research (IPK) OT Gatersleben Seeland Germany; ^3^ Faculty of Natural Sciences I, Institute for Biochemistry and Biotechnology Martin‐Luther‐University Halle‐Wittenberg Halle (Saale) Germany; ^4^ Fraunhofer Institute for Microstructure of Materials and Systems IMWS Halle (Saale) Germany; ^5^ Department Biology, Molecular Plant Physiology Marburg University Marburg Germany; ^6^ Center for Synthetic Microbiology (SYNMIKRO), Marburg University Marburg Germany; ^7^ Faculty of Life Sciences, Department of Biotechnology Ritsumeikan University Kusatsu Japan

**Keywords:** cell‐wall architecture, cell‐wall pore‐size, fungal virulence, hemibiotrophy, PAMP‐triggered immunity, α‐1,3‐glucan

## Abstract

Although α‐1,3‐glucan represents a major polymer in cell walls of filamentous plant pathogenic fungi, its role in vegetative and pathogenic development is poorly understood. The maize anthracnose fungus *Colletotrichum graminicola* differentiates elaborate appressoria on the leaf surface and exhibits a hemibiotrophic lifestyle after host invasion. Promoter‐eGFP‐fusion analyses of all three α‐1,3‐glucan synthase (*AGS*) genes, designated as *AGS1*, *AGS2*, and *AGS3*, suggest that *AGS3* is expressed in vegetative and pathogenic hyphae, whereas expression of *AGS1* and *AGS2* was not, or barely, detectable. Targeted gene deletions yielded single and double deletion mutants, as well as triple mutants, which lacked the entire repertoire of α‐1,3‐glucan synthase genes. Conidia of mutants lacking *AGS3* developed multiple germ tubes, and vegetative hyphae had cell walls containing strongly reduced amounts of alkali‐soluble glucose, showed hyperbranching, reduced growth rates on all substrata tested, and hypersensitivity to the cell wall‐perturbing agents Caspofungin and Nikkomycin Z. Moreover, mutants lacking *AGS3* exhibited strong virulence defects and, intriguingly, biotrophic hyphae of these mutants that differentiated *in planta* were severely misshapen. Increased cell wall pore diameters, increased mobility of uncharged dextran‐FITC conjugates through cell walls of *AGS3*‐deficient mutants and lack of PAMP‐triggered immune responses in maize leaves inoculated with these mutants suggest that α‐1,3‐glucan does not play a role in masking of fungal cell walls and immune evasion, but rather controls mobility of fungal cell wall‐modifying enzymes and cell wall integrity.

## Abbreviated Summary

The role of the major fungal cell wall polymer α‐1,3‐glucan in plant infection is poorly understood. Deletion mutants of the maize anthracnose fungus Colletotrichum graminicola lacking all three α‐1,3‐glucan synthase genes differentiated severely distorted biotrophic hyphae in planta, and virulence was strongly reduced. Reduced virulence was not due to initiation of PAMP‐triggered immunity in maize leaves suggesting that α‐1,3‐glucan does not play a role in immune evasion, but rather in controlling fungal cell wall integrity.

Deletion of genes encoding α‐1,3‐glucan synthases of *Colletotrichum graminicola* caused an increased mobility of molecules within cell walls and compromised cell wall rigidity. Biotrophic hyphae were distorted (arrow), and fungal virulence was strongly reduced, indicating that α‐1,3‐glucan is required for correct cell wall architecture and the infection process.

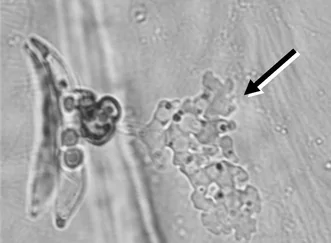

## 1 Introduction

The fungal cell wall determines the shape of cells, protects them from detrimental environmental factors (Gow et al. 2017; Latgé 2007; Wessels 1993; Bowman and Free 2006), and represents the front line in the interaction with competing microorganisms and/or, in the case of pathogenic fungi, with the host (Geoghegan et al. 2017; Pinto et al. 2025). Polysaccharides account for more than 90% of the cell wall of filamentous fungi and have classically been divided into alkali‐soluble and alkali‐insoluble polymers, the latter of which were thought to be determinants of cell wall rigidity (Fontaine et al. 2000). In the filamentous fungus *Aspergillus fumigatus* , a facultative pathogen of humans, enzymatic digestion of vegetative hyphae with recombinant endo‐β‐1,3‐glucanase and chitinase, and subsequent analysis of the resulting fragments by various chromatographic methods, uncovered covalent linkages between mixed‐linked β‐1,3/1,4‐glucan, chitin, and of fungal‐type galactomannan to the non‐reducing end of β‐1,3‐glucan chains (Latgé et al. 2017; Latgé 2007; Fontaine et al. 2000). Alkali‐soluble α‐1,3‐glucan was neither considered a structural polymer nor a determinant of cell wall rigidity. However, chemical and enzymatic methods may fail to reveal the true polymer interconnections due to their destructive nature. More recently, solid‐state NMR spectroscopy, assisted by dynamic nuclear polarization and glycosyl linkage analyses, revealed a high‐resolution model of the native *A. fumigatus* cell wall, with chitin and α‐1,3‐glucan forming a hydrophobic scaffold (Kang et al. 2018). Magic‐angle spinning‐dynamic nuclear polarization (MAS‐DNP) uncovered that 8 out of the 17 cross peaks between chitin and α‐1,3‐glucan were strong interactions, suggesting tight packaging of chitin and α‐1,3‐glucans. Moreover, these studies revealed that the rigid chitin‐α‐1,3‐glucan core is surrounded by a hydrated matrix of diversely linked β‐glucans and capped by a highly mobile shell of glycoproteins and a small, yet significant fraction of α‐1,3‐glucan (Kang et al. 2018). Other studies have hypothesized that α‐1,3‐glucan may be involved in spatially shielding conserved polymers such as chitin, branched β‐glucan, or fungal‐type galactomannan. Thus, α‐1,3‐glucan was thought to contribute to avoiding the release of pathogen‐associated molecular patterns (PAMPs) and to interfere with activation of innate immune responses (Gust et al. 2017; Chaliha et al. 2018). Indeed, α‐1,3‐glucan is present in the outermost layer of the *Histoplasma capsulatum* yeast cell wall, as demonstrated by immune‐electron microscopy, and conceals immune‐stimulatory β‐glucans from detection by host phagocytic cells (Rappleye et al. 2007). In contrast, the conidial surface of *A. fumigatus* is covered by an immunologically inert hydrophobin rodlet layer (Aimanianda et al. 2009). In α‐1,3‐glucan‐deficient triple mutants of *A. fumigatus* , however, extensive structural modification of the conidial cell wall resulted in reduced retainment of a surprisingly large number of proteins, which formed an amorphous glycoprotein layer covering the hydrophobin rodlets. NaCl treatment of conidia of α‐1,3‐glucan‐deficient mutants removed at least 34 proteins, and the NaCl extract, like conidia of the triple mutant, induced the expression of pro‐inflammatory TNFα cytokine (Beauvais et al. 2013). These data suggest that in *A. fumigatus* conidial α‐1,3‐glucan was not involved in preventing immune recognition directly but affected virulence by altering retention and surface‐exposition of innate immune stimulators (Beauvais et al. 2013). These examples show that in distinct mammalian pathogens exhibiting different lifestyles, α‐1,3‐glucan is an important determinant of virulence, but the mechanisms of how this polymer interferes with host defense may differ significantly across pathogens and cell types.

In comparison with mammalian pathogens, the role of α‐1,3‐glucan in cell wall function and virulence has been less intensively studied in plant pathogenic fungi. Interestingly, removal of alkali‐soluble materials from the surface of in vitro differentiated infection structures of the broad bean rust fungus *Uromyces viciae‐fabae* resulted in surface‐exposure of PAMP‐active chitin, as indicated by strong wheat germ agglutinin (WGA) labelling. As these in vitro differentiated infection structures resemble pathogenic hyphae that grow in the intercellular space of host leaves and are thus in direct contact with host cells, surface‐localized alkali‐soluble α‐1,3‐glucan may indeed interfere with PAMP detection and may also have an immune‐concealing role in this obligate biotroph (Freytag and Mendgen 1991). Correspondingly, immuno‐ and lectin‐labelling showed that β‐1,3‐glucan and chitin were hardly detectable prior to, but were visible after treatment of the cell wall of pathogenic hyphae of the rice blast fungus *Magnaporthe oryzae* with α‐1,3‐glucanase (Fujikawa et al. 2009). Supporting the assumption that α‐1,3‐glucan appositions have a shielding function, mutants of this fungus lacking the single‐copy α‐1,3‐glucan synthase gene *AGS1* showed increased susceptibility to treatment with chitinases and β‐1,3‐glucanases (Fujikawa et al. 2012). Interestingly, in contrast to the cell wall model derived from solid‐state NMR data hyphae of *A. fumigatus* (Kang et al. 2018), immuno‐gold‐labelling of infection hyphae of *M. oryzae* with the α‐1,3‐glucan‐specific monoclonal mouse IgMγ antibody MOPC‐104E showed some increase in the density of gold particles towards the outer cell wall surface (Fujikawa et al. 2009). If α‐1,3‐glucan would indeed have a shielding function, it would be expected that massively increased transcript abundances of classical PAMP‐response genes would occur in the host plant inoculated with the *M. oryzae* Δ*ags1* mutant. The enormity of a PAMP response was highlighted in maize leaves inoculated with a mutant of the maize anthracnose fungus *Colletotrichum graminicola* overexpressing the β‐1,3‐glucanase gene *GLS1* during initial biotrophic development, in which genes encoding PR‐proteins, i.e., nine β‐1,3‐glucanase‐, a chitinase‐, a thaumatin‐, ten cell wall peroxidase‐, two cellulose synthase‐, and four terpene synthase‐genes showed dramatically increased transcript abundances. One β‐1,3‐glucanase gene exhibited more than 1000‐fold, and two others, together with two terpene synthase genes, showed a 150‐fold increase in transcript abundances, as compared to leaves inoculated with the wildtype strain (Oliveira‐Garcia and Deising 2013). Similar increases in transcript abundances were also observed in plants inoculated with mutants of the maize anthracnose fungus overexpressing the key β‐1,6‐glucan synthase genes *KRE5* and *KRE6* (Oliveira‐Garcia and Deising 2016a). For comparison, Fujikawa et al. (2012) reported that rice plants inoculated with the *M. oryzae* Δ*ags1* mutant showed only 1.9‐, 2.3‐, and 1.7‐fold increased expression of the PR genes *PR1a*, *PR3*, and *PBZ1*, respectively, as compared to rice plants inoculated with the wildtype strain. In these experiments transcripts had been extracted at 10 h post inoculation (hpi), a time point at which the fungus had neither differentiated an infection cell called an appressorium on the plant cuticle, nor had infection hyphae developed inside the plant cell (Fujikawa et al. 2012). Transcript data from later stages of infection, i.e., after host invasion, were not reported. Moreover, microscopy data showed that infection structures of the Δ*ags1* mutant formed on the rice cuticle lysed before invading the host plant (Fujikawa et al. 2012). Cell wall lysis caused by enzymes such as glucanases or chitinases is unlikely, as the prime antimicrobial defense compounds deposited by glandular trichomes, hydathodes, or resin ducts, are secondary metabolites rather than PR proteins, as thoroughly described in a recent review (Ossola and Farmer 2024). The only defense proteins deposited onto the plant surface by glandular trichomes are the phylloplanins, which interfere with spore germination, but not with hyphal integrity (Ossola and Farmer 2024; Shepherd et al. 2005).

Taken together, the data presented by Fujikawa et al. (2012) suggest, but do not conclusively show, a role of α‐1,3‐glucan in interference with innate immunity of plants. Therefore, the role of this polymer remains poorly understood in plant pathogenic fungi and may vary depending on the fungal species, lifestyle, and/or developmental stage.

Here we report on the role of three AGS genes and of α‐1,3‐glucan in the maize anthracnose and stalk rot fungus *C. graminicola* (Bergstrom and Nicholson 1999), which, like *M. oryzae* , differentiates a pressurized appressorium on the host surface and exhibits a hemibiotrophic lifestyle (O'Connell et al. 2012; Horbach et al. 2011; Bastmeyer et al. 2002; Bechinger et al. 1999). Our studies show that α‐1,3‐glucan is evenly distributed across the hyphal cell wall and is not enriched at the outer surface. Fluorescence‐imaging and Fluorescence‐Recovery‐After‐Photobleaching (FRAP) experiments showed that this polymer controls cell wall permeabilities of molecules of ≥ 20 kDa, and employment of Congo Red and Atomic Force Microscopy indicated a role in the integrity of cell walls of vegetative hyphae and appressoria, respectively. Expression of *AGS3* is primarily induced in biotrophic hyphae. Biotrophic hyphae of all mutants lacking *AGS3* lyze in intact as well as in boiled host epidermal cells, indicating autolysis due to severe cell wall defects. α‐1,3‐glucan is required for full virulence of *C. graminicola* on maize leaves, and PAMP‐response genes identified previously are not induced in leaves inoculated by the triple mutant lacking all *AGS* genes. We conclude that α‐1,3‐glucan is required for cell wall function and full virulence, but does not exhibit a PAMP‐concealing function.

## 2 Results

### 2.1 α‐1,3‐Glucan Is Required for Cell Wall Function in Vegetative Hyphae of *C. graminicola*

Genome mining (https://mycocosm.jgi.doe.gov/Colgr1/Colgr1.home.html) revealed that the maize anthracnose and stalk rot fungus *C. graminicola* possesses three α‐1,3‐glucan synthase‐coding genes, designated as *AGS1* (accession number GLRG_07192.1), *AGS2* (GLRG_09823.1) and *AGS3* (GLRG_10929.1). The genes are 7767, 8261, and 7232 bp in size and harbor 8, 6 and 3 introns, respectively (Figure 1A). The proteins Ags1, Ags2 and Ags3 consist of 2375, 2402, and 2351 amino acids (aa), respectively. All three α‐1,3‐glucan synthase proteins harbor an N‐terminal domain with similarity to the catalytic domain of α‐amylases and a C‐terminal domain with similarity to GlgA‐type superfamily glycogen synthases (Figure 1B). Ags1 contains a central GTB‐type superfamily glycosyl transferase‐like domain, whereas Ags2 and Ags3 possess a DULL1‐like GTI glycogen synthase domain (Figure 1B). Comparisons of the entire amino acid sequences indicates that Ags3 of *C. graminicola* is most closely related to Ags1 of the Arabidopsis pathogen *Colletotrichum higginsianum* and Ags1 of *M. oryzae* , sharing 53.8% and 51.6% amino acid sequence identity, respectively. The genome of the fission yeast *Schizosaccharomyces pombe* five genes encoding α‐1,3‐glucan synthases in the latter species, that are designated as SpAgs1 and SpAgs12, and SpMok11–13. Not only yeasts, but also filamentous ascomycetes harbor distinct numbers of α‐1,3‐glucan synthase‐encoding genes. The *M. oryzae* genome contains a single gene, the genome of *C. higginsianum* contains two genes, and the genomes of *A. fumigatus* and *C. graminicola* have three α‐1,3‐glucan synthase‐encoding genes. The α‐1,3‐glucan synthases of yeasts and filamentous fungi localize to distinct branches of the phylogenetic tree (Figure 1C).

**FIGURE 1:**
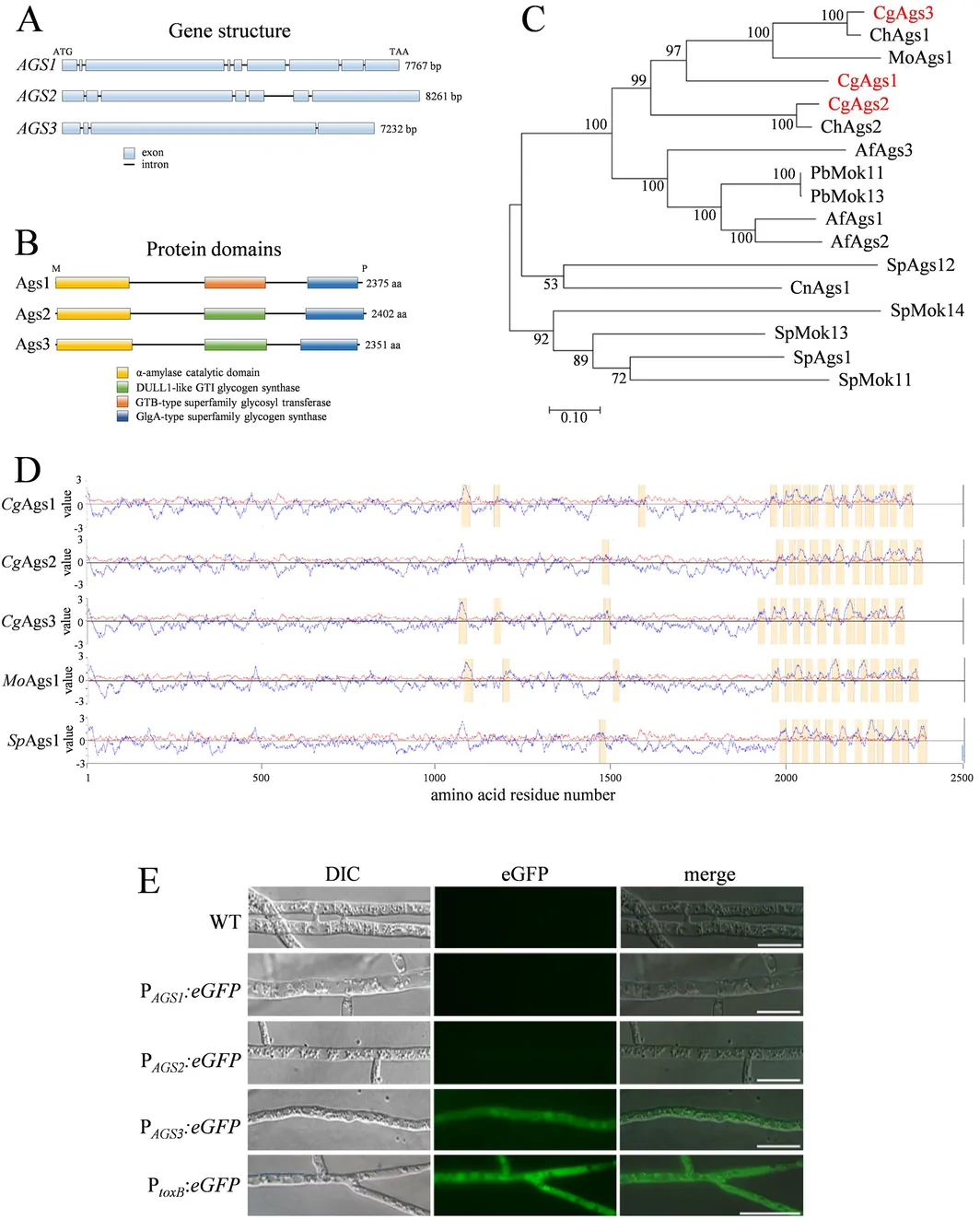
The *C. graminicola* genome harbors three α‐1,3‐glucan synthase genes, of which only one is expressed in vegetative hyphae. (A) Structure of *AGS1*, *AGS2*, and *AGS3*. Blue bars represent exons, black lines represent introns. The size of individual genes is given in base pairs. (B) Protein domains of Ags1, Ags2, and Ags3, as predicted by NCBI conserved Domain Search and UniProt. Protein sizes are given in amino acids. (C) Maximum likelihood tree indicating the phylogenetic relatedness of the three *C. graminicola* α‐1,3‐glucan synthase proteins with related proteins from other filamentous fungi and yeasts. Sequences were aligned using the CLUSTAL W program, and the phylogenetic tree was constructed using the Neighbor‐Joining method. Bootstrap values are % of 1000 replicates. Accession numbers of α‐1,3‐glucan synthase proteins are given in the Materials and Methods section. (D) Hydropathy plots predict 12 C‐terminal membrane‐spanning α‐helices (beige bars) in Ags1, Ags2, and Ags3 of *C. graminicola* (*Cg*Ags1–Ags3), Ags1 of *M. oryzae* (*Mo*Ags1) and *S. pombe* Ags1. *Cg*Ags1, *Cg*Ags3, and *Mo*Ags1 show three central transmembrane domains, *Cg*Ags2 and *Sp*Ags1 possess only one. (E) DIC and eGFP micrographs of the *C. graminicola* WT strain and transformants harboring the promoter‐*eGFP* constructs P_*AGS1*_ *:EGFP*, P_*AGS2*_ *:EGFP*, and P_*AGS3*_ *:EGFP*. A *C. graminicola* transformant expressing the constitutive P_*toxB*_:*EGFP* construct served as control. Size bars represent 20 μm.

Hydropathy plots, as created using the TC database with a standard window size of 19 amino acids (http://www.tcdb.org/progs/?tool=hydro), show that all AGS proteins compared here have 12 predicted C‐terminal transmembrane α‐helices, suggesting membrane‐association of the enzymes. Ags1 and Ags3 of *C. graminicola* , like Ags1 of *M. oryzae* , have three additional central transmembrane regions and are distinct from Ags2 of *C. graminicola* and Ags1 of the yeast *S. pombe* (Figure 1D).

In order to investigate the expression of *AGS1*, *AGS2*, and *AGS3* of *C. graminicola* , we generated *AGS* promoter‐*eGFP* fusion strains (Figure S1). Analysis of gene expression employing *eGFP* fusions allows us to visualize cell‐specific expression even in cells that are differentiated in a non‐synchronized manner, e.g., in infection structures of the hemibiotrophic fungus *C. graminicola* . *eGFP* fusions allowed demonstrating that expression of the β‐glucan biosynthesis genes *GLS1*, *KRE5*, and *KRE6* was specifically down‐regulated in biotrophic infection structures to circumvent PAMP detection (Oliveira‐Garcia and Deising 2013, 2016a, 2016b). In cells that are poorly synchronized, RNA‐Seq or RT‐qPCR would not allow resolving stage specificity (see O'Connell et al. 2012). P_*AGS1*_, P_*AGS2*_, and P_*AGS3*_ promoter‐*eGFP* fusions (Figure S1A) were transformed into protoplasts generated from oval conidia of the *C. graminicola* WT strain, and distinct and independent transformants harboring single copies of randomly integrated promoter‐eGFP cassettes were identified by genomic Southern blot analyses (Figure S1B; asterisks). Independent transformants were compared to the WT strain with respect to colony morphology, hyphal growth, and sporulation rates on oatmeal (OMA) and potato dextrose agar (PDA) (Figure S1C–E), and with regard to virulence on non‐wounded and wounded maize ( *Zea mays* cv. Mikado) leaves (Figure S1F). No statistical differences were found in any of the parameters tested, indicating that integrations did not affect genes required for vegetative and pathogenic development of these transformants. Interestingly, only vegetative hyphae of strains harboring a P_*AGS3*_:*eGFP* construct, but not the P_*AGS1*_:*eGFP*‐ and P_*AGS2*_:*eGFP*‐containing strains, exhibited eGFP fluorescence, indicating that *AGS3*, but not *AGS1* and *AGS2*, is strongly expressed in vegetative hypha (Figure 1E). As expected, hyphae of a *C. graminicola* strain expressing eGFP under the control of the strong constitutive *TOXB* promoter (P_*TOXB*_) of the wheat pathogen (Andrie et al. 2005; Albarouki and Deising 2013) showed strong fluorescence (Figure 1E; P_*toxB*_:*eGFP*). Hyphae of the non‐transformed WT strain of *C. graminicola* did not auto‐fluoresce under the conditions tested (Figure 1E; WT).

In order to determine the roles of the three *AGS* genes, and their products, of *C. graminicola* , seven distinct α‐1,3‐glucan synthase deletion mutants, i.e., the single deletion mutants Δ*ags1*, Δ*ags2*, and Δ*ags3*, as well as all combinations of multiple mutants, including double and triple deletion mutants, were generated (Figure S2).

Chemical analyses showed that the alkali‐soluble fraction (ASF) of the cell wall was smaller in the Δ*ags1,2,3* triple mutant than in the WT strain (Figure 2A). It is conceivable that this is due to lower glucose content (*p* < 0.05) in the ASF of cell walls of the Δ*ags1,2,3* triple mutant compared with that of the WT strain (Figure 2B; ASF). Interestingly, the galactose content was increased in the ASF of the triple mutant (Figure 2B; ASF). Fungal‐Type galactomannan is present in the mobile phase of the cell wall, as shown by high‐resolution solid‐state NMR studies (Chakraborty et al. 2021) and contributes to cell wall rigidity (Onoue et al. 2018), increased galactose contents in the ASF of the Δ*ags1,2,3* mutant may represent a compensatory response. In the alkali‐insoluble fraction, differences in the sugar composition between the WT and the triple mutant were minor and statistically not significant (Figure 2B, AIF).

**FIGURE 2:**
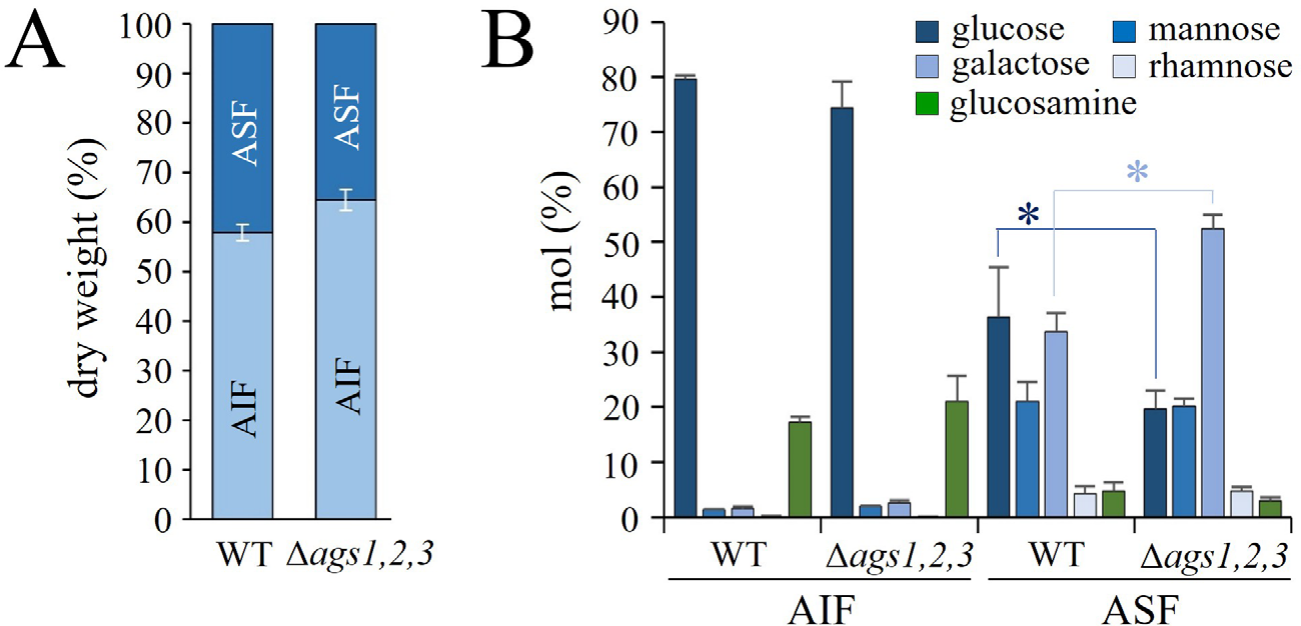
Mycelial cell wall composition of the *C. graminicola* WT and α‐1,3‐glucan synthase‐deficient triple mutant Δ*ags1,2,3*. (A) Alkali‐insoluble and alkali‐soluble fractions (AIS and ASF) indicate loss of alkali‐soluble α‐1,3‐glucan in Δ*ags1,2,3* strains. Fractions are % of cell wall dry weight. (B) Hexose composition of AIF and ASF of WT and Δ*ags1,2,3* strains. Error bars show standard deviations from four biological replicates and asterisks indicate statistical significance at *p* < 0.05 in a two‐way ANOVA with a Holm‐Sidak post hoc test.

Growth assays on all substrata tested revealed significant radial growth defects in Δ*ags3* mutants and in all higher order mutants lacking the *AGS3* gene (Figure 3A,B), which supports the fluorescence data showing that *AGS3* was the most strongly expressed *AGS* gene in vegetative hyphae. Growth rates of the Δ*ags1* and Δ*ags2* single mutants did not differ significantly from those of the WT strain (Figure 3A,B). As the Δ*ags1,3* (Figure 3A,B; MMA) and Δ*ags2,3* double mutants (Figure 3A,B; all media) showed more severe growth phenotypes than the Δ*ags3* single mutant, it can be concluded that *AGS1* and *AGS2* also play minor roles in vegetative growth. Low expression rates of these genes may have escaped detection by fluorescence microscopy (Figure 1E; P_*AGS1*_:*eGFP* and P_*AGS2*_:*eGFP*). Differential Interference Contrast (DIC) microscopy revealed that in all of the *AGS*‐deficient mutants tested, the rate of germ tube formation per conidium (Figure S3), as well as that in all mutants lacking *AGS3* hyphal branching, was strongly increased (Figure 3C,D). Intriguingly, increased branching rates have previously been associated with reduced cell wall rigidity (Richard et al. 2002; Oliveira‐Garcia and Deising 2016b).

**FIGURE 3:**
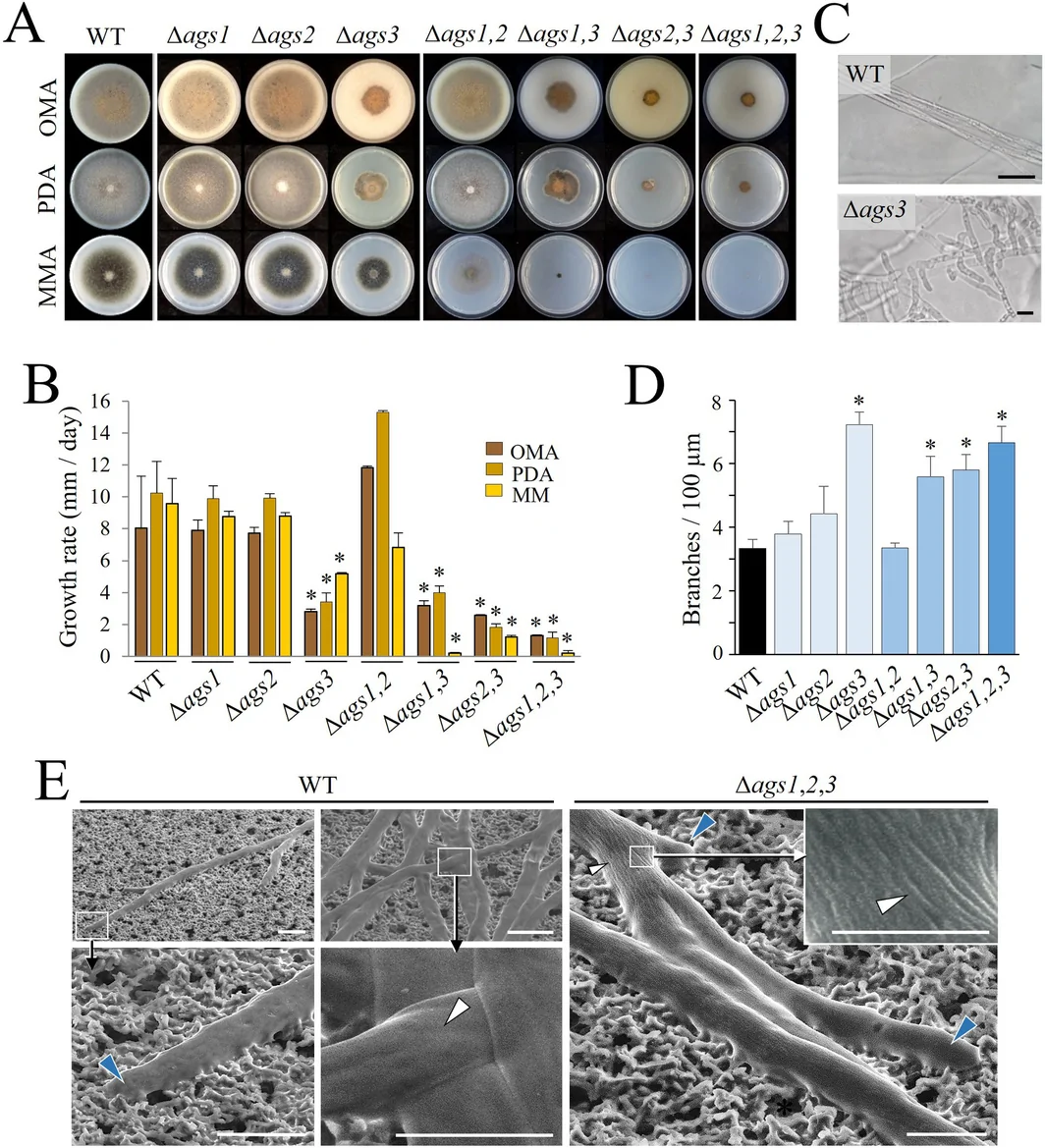
Growth and characteristics of vegetative hyphae of the *C. graminicola* WT strain, and of single and multiple *AGS* deletion mutants. (A) Growth and colony phenotype on oat meal agar (OMA), potato dextrose agar (PDA), and minimum medium agar (MMA). (B) Quantitative growth rates of WT and *AGS* mutants on three different substrata. (C) Micrographs showing unbranched hyphae of the WT and highly branched hyphae of the Δ*ags3* mutant. (D) Quantification of branching rates of hyphae of the WT and of all *AGS* deletion mutants. (E) ESEM micrographs of vegetative hyphae of WT and the Δ*ags1,2,3* triple mutant. Blue arrowheads indicate hyphal tips, white arrowheads highlight surfaces of older hyphal regions. Boxed areas are shown at higher magnification in the lower panels (WT) or as insert (Δ*ags1,2,3*). Note the smooth hyphal surface of WT and the wrinkled surface of the Δ*ags1,2,3* mutant (arrowheads). Size bars in (C, E) represent 5 μm. (B, D) show data of three biological replicates; error bars indicate standard deviations of the mean. Asterisks indicate statistical significance at *p* < 0.05.

As previous work has shown that hyphal or conidial surface properties are altered in mutants lacking α‐1,3‐glucan, either due to altered retention of molecules in the cell wall or due to a lack of α‐1,3‐glucan appositions (Beauvais et al. 2013; Rappleye et al. 2007), we employed Environmental Scanning Electron Microscopy (ESEM) to examine the surface texture of vegetative hyphae of the WT strain and of the Δ*ags1,2,3* triple mutant growing on a Nylon membrane (Hybond‐N+; Amersham Pharmacia Biotech, Freiburg) covering the PDA (Figure 3E). Hyphal tips of both the WT and the Δ*ags1,2,3* triple mutant (Figure 3E; blue arrowheads) showed rather smooth surfaces. The smooth surface extended to older hyphae in the WT strain (Figure 3E, WT, white arrowhead). In contrast, the surface texture of older hyphae of the Δ*ags1,2,3* triple mutant showed a wrinkled surface texture (Figure 3E, Δ*ags1,2,3*, white arrowhead). These observations suggest that surface appositions occur on the hyphal surface of the Δ*ags1,2,3* mutant due to reduced retention of materials within the cell wall. This has also been reported for hyphae of α‐1,3‐glucan‐defective mutants of *A. fumigatus* (Beauvais et al. 2013).

The role of the *AGS* gene products in cell wall function was further investigated by inoculating the WT and the Δ*ags1,2,3* mutant onto PDA and PDA amended with the β‐1,3‐glucan biosynthesis inhibitor Caspofungin (Feldmesser et al. 2000; Serfling et al. 2007) or with the chitin biosynthesis inhibitor Nikkomycin Z (Wu et al. 2022; Werner et al. 2007) (Figure S4). On PDA, the WT strain showed regular hyphal growth, with occasional branching and intact hyphal tips (Figure S4, right panel, black arrowheads). The triple mutant produced shorter, pigmented cells (Figure S4, right panel, black arrow), and occasional slightly swollen hyphal tips (Figure S4, right panel, white arrow). Caspofungin clearly affected colony growth of the WT as well as that of the Δ*ags1,2,3* mutant (Figure S4, left panel), but this drug did not visibly affect the hyphae of the WT strain, as indicated by intact hyphal tips (Figure S4, right panel; black arrowheads). Interestingly, hyphae of the Δ*ags1,2,3* mutant occasionally showed lysis at the hyphal tips growing at the margin of the colony (Figure S4, right panel; white arrowhead). In the presence of Congo Red (data not shown), which interferes with β‐glucan assembly (Kopecká and Gabriel 1992) and diminishes the interaction between β‐1,3‐glucan and chitin (Kang et al. 2018), hyphae of the Δ*ags1,2,3* mutants showed slightly stronger hyphal tip swellings than those of the WT strain. While Nikkomycin Z showed only minor effects on WT colony growth, it severely affected growth of the Δ*ags1,2,3* mutant (Figure S4, left panel). Microscopy showed massive ballooning of WT hyphae (Figure S4, right panel, white arrows) and some pigmentation (Figure S4, right panel, black arrow). Also, in the triple mutant swellings occurred and severe pigmentation was observed (Figure S4, right panel, white and black arrows). Moreover, the Δ*ags1,2,3* mutant formed setae (Figure S4, right panel, thin white arrow) and large numbers of conidia (Figure S4, right panel, thin black arrow).

These inhibitor studies including the WT and the Δ*ags1,2,3* mutant suggest that all structural polymers, i.e., α‐1,3‐glucan, chitin and β‐1,3‐glucan are required for cell wall function and vegetative development.

### 2.2 α‐1,3‐Glucan is Evenly Distributed Across the Cell Wall of Vegetative Hyphae and Present on the Hyphal Surface of *C. graminicola*

In order to analyze the localization of α‐1,3‐glucan in hyphal cell walls of *C. graminicola* , the murine IgM antibody MOPC 104E was used in combination with a Alexa Fluor 488‐conjugated secondary antibody. As an additional independent probe, the GFP conjugate of the α‐1,3‐glucan‐binding discoidin domain DS1 of *Bacillus circulans* KA‐304 α‐1,3‐glucanase (Suyotha et al. 2013) was employed. Both probes bound to cell walls of intact vegetative hyphae of the WT strain and caused strong fluorescence. In contrast, hyphae of the Δ*ags1,2,3* mutant did not fluoresce, indicating the absence of α‐1,3‐glucan surface exposure (Figure 4A). To investigate whether α‐1,3‐glucan is primarily surface‐associated or also present across the entire cell wall, ultra‐thin cell wall cross‐sections were probed with MOPC 104E and a gold‐conjugated secondary antibody. The WT hyphae showed significant immune‐gold labelling across the entire cell wall (Figure 4B; WT, white arrowheads). Individual gold particles were detected on the outer hyphal surface (Figure 4B, black arrow), supporting detection of α‐1,3‐glucan by externally applied DS1‐GFP and MOPS 104E probes. Quantitative analysis revealed that gold particles were evenly distributed across the cell wall and not primarily associated with the outer hyphal surface (Figure 4C). Hyphal cell walls of the Δ*ags1,2,3* triple mutant were not labelled (Figure 4B; Δ*ags1,2,3*).

**FIGURE 4:**
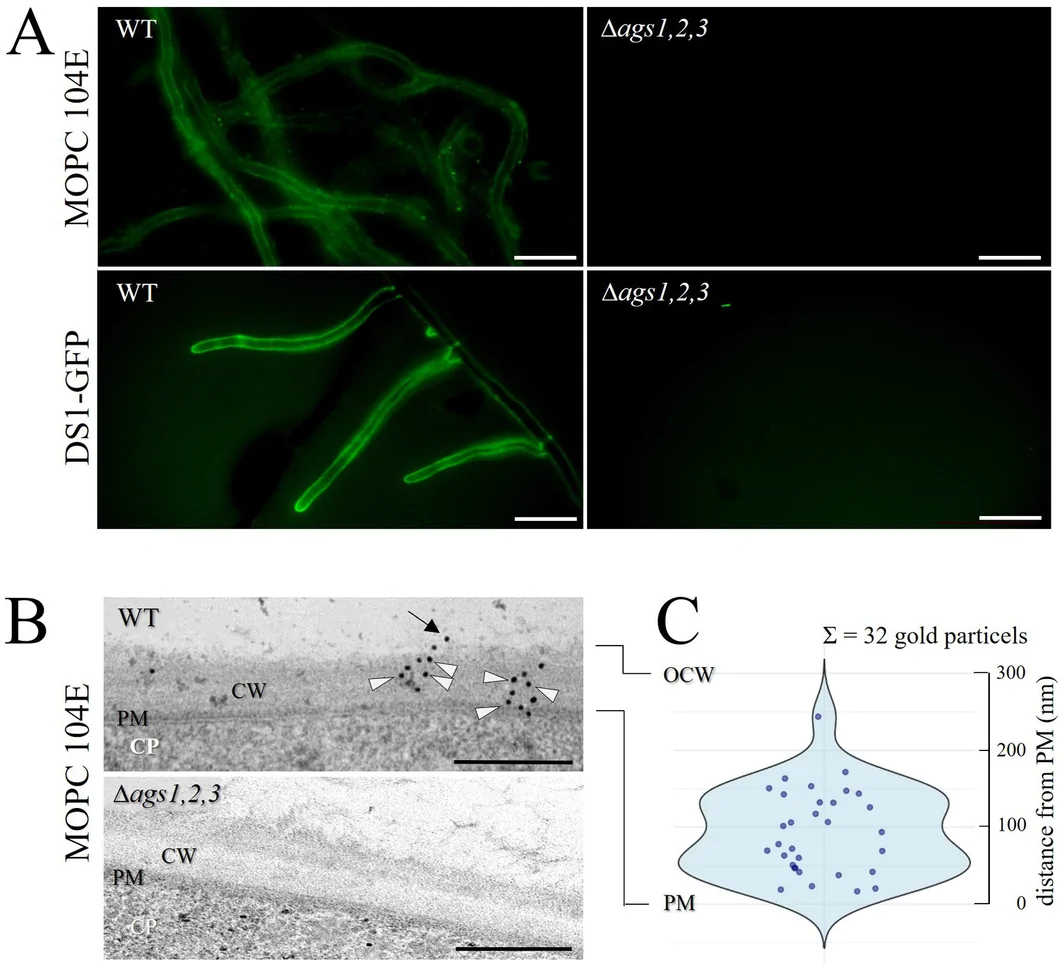
α‐1,3‐glucan is detectable on the hyphal surface and is evenly distributed across sections of vegetative hyphae of the WT strain of *C. graminicola* , but is absent from the Δ*ags1,2,3* triple mutant. (A) Labelling of intact hyphae of the *C. graminicola* WT strain with two independent probes, i.e., MOPC 104E and DS1‐GFP. Size bars represent 10 μm. (B) Transmission electron micrographs of immuno‐gold‐labeled hyphal cross sections of the WT strain shows gold particles across the entire hyphal cell wall (white arrowheads) and on the surface (arrow). Size bars represent 200 nm. CP, cytoplasm; CW, cell wall; OCW, outer cell wall; PM, plasma membrane. (C) Quantitative illustration of distribution of gold particles in 53 images of the WT cell wall shows near‐uniform distribution of gold particles across the hyphal wall. The Δ*ags1,2,3* mutant shows no α‐1,3‐glucan labelling.

### 2.3 α‐1,3‐Glucan Restricts Cell Wall Pore Size and Mobility of Molecules

Restriction of cell wall pore sizes by α‐1,3‐glucan would restrict the freedom of movement of cell wall‐modifying enzymes and, hence, the integrity of fungal cell walls (Adams 2004; Samalova et al. 2017). Increased branching rates (Figure 3D), swelling of hyphal tips (Figure S4B), and conidia germinating with more than one germ tube (Figure S3) in mutants lacking *AGS3* may be taken as an indication of reduced cell wall rigidity and suggest the investigation of whether α‐1,3‐glucan has a role in the control of cell wall pore size.

In order to compare cell wall pore diameters of the WT strain with those in the Δ*ags1,2,3* triple mutant lacking α‐1,3‐glucan, we employed Fluorescence Imaging and FRAP assays, using uncharged dextran‐FITC conjugates with mean molecular masses of 4, 20, and 70 kDa, corresponding to radii of approx. 1.5, 3.2 and 6 nm, respectively (Figure S5A,B). Germinating conidia of *C. graminicola* differentiate a short germ tube, which, in turn, forms an appressorium on hydrophobic substrata such as the plant cuticle or artificial polyethylene sheets. During pre‐invasive development, the cytoplasm moves into the appressorium and is captured in this cell by septation at the germ tube‐appressorium border, leaving the conidium empty. These empty cells are ideally suited for fluorescence imaging analyses.

High resolution confocal microscopy showed that fluorescent dextran molecules of 4 kDa, corresponding to an average radius of 1.4 nm, were able to pass conidial cell walls of both WT and Δ*ags1,2,3* strains resulting in fluorescing conidial lumina (Figure 5A, 4 kDa, arrowheads). By contrast, neither the 20 kDa nor the 70 kDa fluorescent dextran probes were able to pass the cell wall of WT conidia, resulting in non‐fluorescing cells (Figure 5A; WT, co, 20 kDa and 70 kDa). In conidia of the Δ*ags1,2,3* mutant, however, both 20 kDa and 70 kDa dextrans were found intracellularly at lower, but nevertheless clearly detectable concentrations (Figure 5A; compare WT and Δ*ags1,2,3* conidia treated with 20 kDa and 70 kDa probes; asterisks). These fluorescence microscopy studies indicate that molecules corresponding to average radii of 3.3 nm or indeed 6 nm were able to migrate across cell walls of empty conidia of α‐1,3‐glucan‐deficient, but not across those of the WT strain. Moreover, quantitative analysis of the permeability, here defined as the ratio of relative fluorescence intensity per unit area inside versus outside the conidia, confirmed that the fluorescing dextran molecules of all sizes migrated more readily through the cell walls of the Δ*ags1,2,3* triple mutant than through WT cell walls (Figure 5B). While the permeability of the 4 kDa probe in the Δ*ags1,2,3* mutant was nearly comparable to that in the WT, the larger probes (20 kDa and 70 kDa) exhibited more than two‐ and threefold higher permeabilities, as compared to WT (Figure 5B). Diffusion measurements by monitoring the recovery of fluorescence in photobleached regions of the cytoplasm (quantitative FRAP assays) comparably showed that the mobility of 20 kDa and 70 kDa FITC‐labelled dextran molecules is increased in the cell wall of the Δ*ags1,2,3* triple mutant, as compared to the cell wall of the WT strain (Figure 5C). Consistent recovery times (~200 ms) across all molecular weights and samples (Figure S5A,B) suggest that only shorter chains contributed significantly to the mobile fraction, while passage of larger chains through the cell wall is precluded and thus do not add to the internal mobility (Figure S5C). However, the higher permeability of cell walls of the Δ*ags1,2,3* triple mutant allowed the mobile fractions to be slightly increased (Figure 5C). Strengthening the hypothesis that the lack of α‐1,3‐glucan increases the cell wall pore size, we show that a larger number of proteins can be extracted from cell walls of the Δ*ags1,2,3* mutant (Figure 5D). Moreover, the fact that more proteins of a molecular mass of 20–50 kDa can be extracted than larger proteins of a mass between 70 and 130 kDa (Figure 5D) provides further evidence supporting the mobility data shown in Figure 5A–C and suggests that the lack of α‐1,3‐glucan affects cell wall pore size and molecular mobility. However, these experiments do not exclude that other physicochemical characteristics such as changed polarity, adhesion, or fluidity of the cell wall polymers themselves may contribute to mobility of molecules.

**FIGURE 5:**
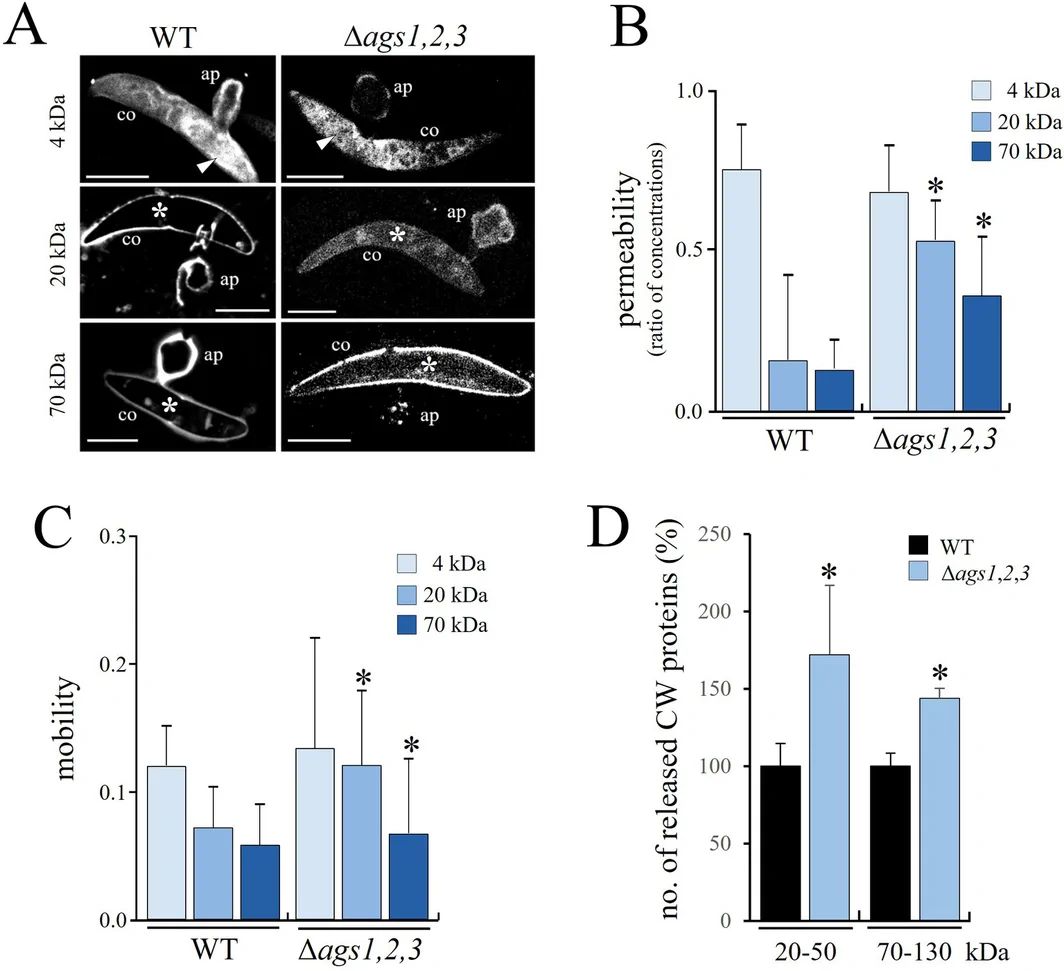
α‐1,3‐glucan reduced cell wall pore sizes and mobility of FITC‐labelled 4, 20, and 70 kDa dextrans in cell walls. (A) FITC‐labelled 4 kDa dextrans are able to cross the conidial cell wall of the WT and the Δ*ags1,2,3* mutant, resulting in fluorescing lumina of conidia (co, white arrowhead). FITC‐labelled 20 and 70 kDa dextrans do not pass the cell walls of WT conidia, resulting in lack of fluorescence in conidia (WT, co, asterisk), but they are able to migrate into conidia of the Δ*ags1,2,3* mutant (Δ*ags1,2,3*, co, asterisk). Note that the fluorescence in conidia of the Δ*ags1,2,3* mutant decreased with increasing molecular masses of dextrans. Size bars represent 10 μm. (B) Permeation of FITC‐labelled 4, 20, and 70 kDa dextrans through conidial cell walls of the WT and the Δ*ags1,2,3* mutant, as calculated from the mean fluorescence intensities of cell walls vs. lumina of conidia. In total, 41 measurements were performed. Error bars are standard deviations; asterisks indicate statistical distinctness from the WT at *p* < 0.05. (C) Quantitative FRAP experiments showing mean mobility values of FITC‐labelled 4, 20, and 70 kDa dextrans in conidial cell walls of the WT and the Δ*ags1,2,3* mutant. In total, 98 FRAP measurements were performed. Error bars represent standard deviation calculated from > 30 measurements. Asterisk on bar representing 20 kDa indicates statistical significance at *p* = 0.10, asterisk on bar representing 70 kDa indicates statistical significance at *p* = 0.13. (D) Relative number of proteins extractable from cell walls of the WT and the Δ*ags1,2,3* mutant. On a percentage basis, more proteins were extractable from the mutant cell walls, with a higher proportion of smaller proteins. Error bars are standard deviations; asterisks indicate statistical distinctness from the WT at *p* < 0.05.

### 2.4 α‐1,3‐Glucan Is Required for Cell Wall Function in Appressoria of *C. graminicola*

In order to invade the host epidermal cell, appressoria of *C. graminicola* develop an enormous turgor pressure of approx. 5.35 MPa (Bechinger et al. 1999), and rigid appressorial cell walls are required to control this turgor pressure (Oliveira‐Garcia and Deising 2013, 2016a). All α‐1,3‐glucan synthase mutants differentiated which were not distinct from WT appressoria, as judged by light microscopy (Figure 6A, ap), but, surprisingly, appressoria of mutants lacking *AGS3* germinated laterally and formed secondary appressoria at high frequency (Figure 6A, ap*,B), indicative of weakened cell walls of primary appressoria. Lateral appressorial germination and formation of secondary appressoria has also been observed in other mutants lacking cell wall biogenesis genes (Groß et al. 2024). Interestingly, environmental scanning electron microscopy (ESEM) showed that appressoria of both WT and Δ*ags1,2,3* strains are comparable at low magnification (ESEM, asterisks), with mucilaginous material (ESEM arrows) previously shown to contain galactosaminogalactan required for appressorial adhesion (Groß et al. 2024). Higher magnification of the appressorial surface suggests, however, that in the α‐1,3‐glucan‐defective Δ*ags1,2,3* mutant, string‐like structures are more deeply embedded into a smooth matrix than in the WT (Figure 6C, ESEM, lower panel). In line with these observations, fibrous structures were clearly detected by surface topography scans using Atomic Force Microscopy (AFM) on the appressorial surface of the WT strain, but these were barely visible on that of the Δ*ags1,2,3* mutant (Figure 6C; AFM, WT, arrow).

**FIGURE 6:**
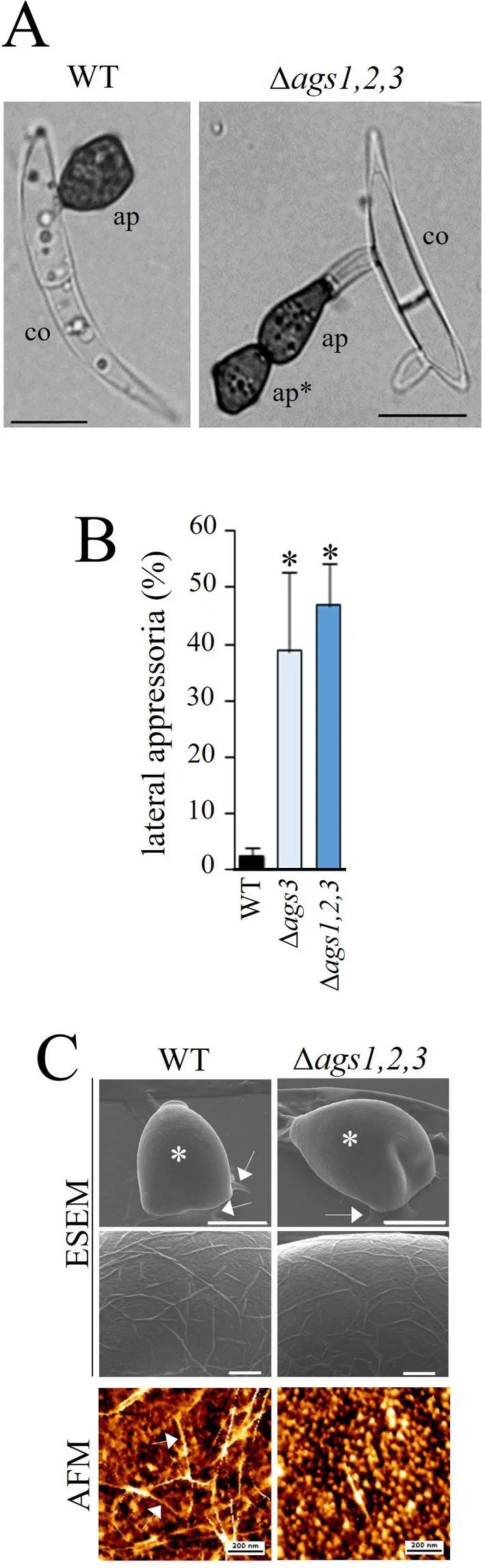
α‐1,3‐glucan is required for appressorial cell wall rigidity and molecular retention. (A) Conidia (co) of the WT strain germinate and differentiate a melanized appressorium (ap) on a hydrophobic surface and remain unaltered, as the polyethylene sheet cannot be penetrated. Appressoria (ap) of the Δ*ags1,2,3* mutant are melanized and phenotypically comparable to those of the WT strain, but they frequently form lateral secondary appressoria (ap*). Size bars represent 10 μm. The photograph was taken at 48 hpi. (B) Quantification of lateral appressoria differentiated by the WT, the Δ*ags3* and the Δ*ags1,2,3* mutants. One‐hundred appressoria of three biological replicates were quantified per genotype. Error bars show standard deviations, asterisks indicate statistically significant distinctness from the WT at *p* < 0.05. (C) ESEM shows that appressoria (asterisks) of the WT and the Δ*ags1,2,3* mutant are phenotypically comparable and form an adhesive mucilage (arrows). Size bars represent 5 μm. On the surface of WT appressoria, strands appear to be embedded in surface material to a lesser extent compared to the Δ*ags1,2,3* mutant (ESEM, lower panel). Size bars represent 200 nm. Confirming these observations, also AFM shows more rigid strands in the WT strain than in the Δ*ags1,2,3* strain. Size bars represent 200 nm.

### 2.5 Ags3 Produces α‐1,3‐Glucan in Biotrophic Infection Structures and Is Required for Full Virulence of the Maize Anthracnose Fungus

To investigate the role of α‐1,3‐glucan synthase genes in plant pathogenesis, infection assays were performed on non‐wounded and wounded maize ( *Zea mays* cv. Mikado) leaf segments (Figure 7A). Of the single deletion mutants, only the Δ*ags3* strains showed strongly reduced anthracnose disease symptoms, while after inoculation of the Δ*ags1,2,3* triple mutant anthracnose symptoms did not occur on non‐wounded leaves (Figure 7A; non‐wounded, Δ*ags3* and Δ*ags1,2,3*). On maize leaves, differences in disease severity were likely not due to compromised appressorial penetration competence of strains lacking *AGS3*, as penetration rates of the WT strain and the Δ*ags1*, Δ*ags2* and Δ*ags3* mutants were statistically not distinct at 48 and 72 h post inoculation (hpi) (data not shown). Upon inoculation of conidia onto wounded leaves, the hemibiotrophic fungus does not differentiate biotrophic hyphae, but rather directly forms destructive necrotrophic hyphae (Oliveira‐Garcia and Deising 2013). The severity of disease symptoms caused by the WT strain on wounded leaves was comparable to that observed on WT‐inoculated non‐wounded leaves (Figure 7A). On wounded leaves, the Δ*ags3* single and the Δ*ags1,2,3* triple mutants caused more pronounced disease symptoms than on non‐wounded leaves; however, symptoms were less severe than those caused by the WT strain (Figure 7A; wounded leaves). These data suggest that *AGS3* is of particular importance during biotrophic development. To validate this hypothesis, we employed *eGFP* promoter fusion strains to visualize expression of the three different α‐1,3‐glucan synthase genes during pathogenesis. As indicated above, all promoter fusion strains used in this study showed WT‐like virulence, indicating that integration of these constructs did not interfere with the infection process (Figure S1F). As observed for vegetative hyphae, only the *AGS3* promoter (P_*AGS3*_) conferred eGFP fluorescence in infected leaves. Intriguingly, P_*AGS3*_:*eGFP* strains showed strong fluorescence specifically in biotrophic hyphae (Figure 7B; P_*AGS3*_:*eGFP*, 48 hpi, arrows), while necrotrophic hyphae showed much weaker, but nevertheless clearly visible, fluorescence (Figure 7B; P_*AGS3*_:*eGFP*, 72 hpi, arrow). Fluorescence in biotrophic and necrotrophic hyphae of strains harboring the P_*AGS3*_:*eGFP* construct was significantly stronger than eGFP fluorescence in P_*AGS1*_:*eGFP* or P_*AGS2*_:*eGFP* strains at any stage of pathogenesis (Figure 7B,C). These data strongly suggested that *AGS3* is of particular importance in biotrophic hyphae. In order to investigate the importance of α‐1,3‐glucan in biotrophic and necrotrophic infection structures, we inoculated conidia of the WT and the α‐1,3‐glucan‐deficient Δ*ags1,2,3* triple mutant onto epidermal cell layers of the alternative host onion (Oliveira‐Garcia and Deising 2013), allowing simple analysis of the infection process by light microscopy. On intact cells of this host, the WT strain formed appressoria, which efficiently invaded the host cell and sequentially differentiated voluminous biotrophic (Figure 7D; WT, vital epidermis, bh) and thin fast‐growing destructive necrotrophic hyphae (Figure 7D; WT, vital epidermis, nh). Like the WT strain, the Δ*ags1,2,3* mutant also formed appressoria and invaded the onion epidermis cells (Figure 7D; Δ*ags1,2,3*, vital epidermis). Surprisingly, however, the biotrophic hyphae showed large lobes and were severely distorted (Figure 7D; Δ*ags1,2,3*, vital epidermis, dbh). To investigate whether distortion of biotrophic hyphae of the Δ*ags1,2,3* mutant was caused by the activity of host PR‐proteins such as chitinases or β‐glucanases, due to lack of hyphal masking by α‐1,3‐glucan, or by fungal cell wall‐modifying enzymes, which gained increased mobility in cell walls lacking α‐1,3‐glucan, we inoculated conidia of the WT and of the Δ*ags1,2,3* mutant onto boiled onion epidermis layers, in which enzymes had been heat‐inactivated. Intriguingly, even in heat‐treated onion cells, the Δ*ags1,2,3* mutant, but not the WT strain, formed severely distorted biotrophic hyphae (Figure 7D; Δ*ags1,2,3*, compare dbh vital epidermis in vital and boiled host). Occasionally distorted biotrophic hyphae of the Δ*ags1,2,3* mutant were able to conduct the biotrophy‐to‐necrotrophy switch, and these necrotrophic hyphae growing out of the distorted biotrophic hyphae were morphologically indistinguishable from those differentiated by the WT strain (Figure 7D; Δ*ags1,2,3*, right panel, white arrow). Distorted biotrophic hyphae were never observed in the WT strain, but a significant fraction of these was found for the Δ*ags3* and Δ*ags1,2,3* mutants (Figure 7E).

**FIGURE 7:**
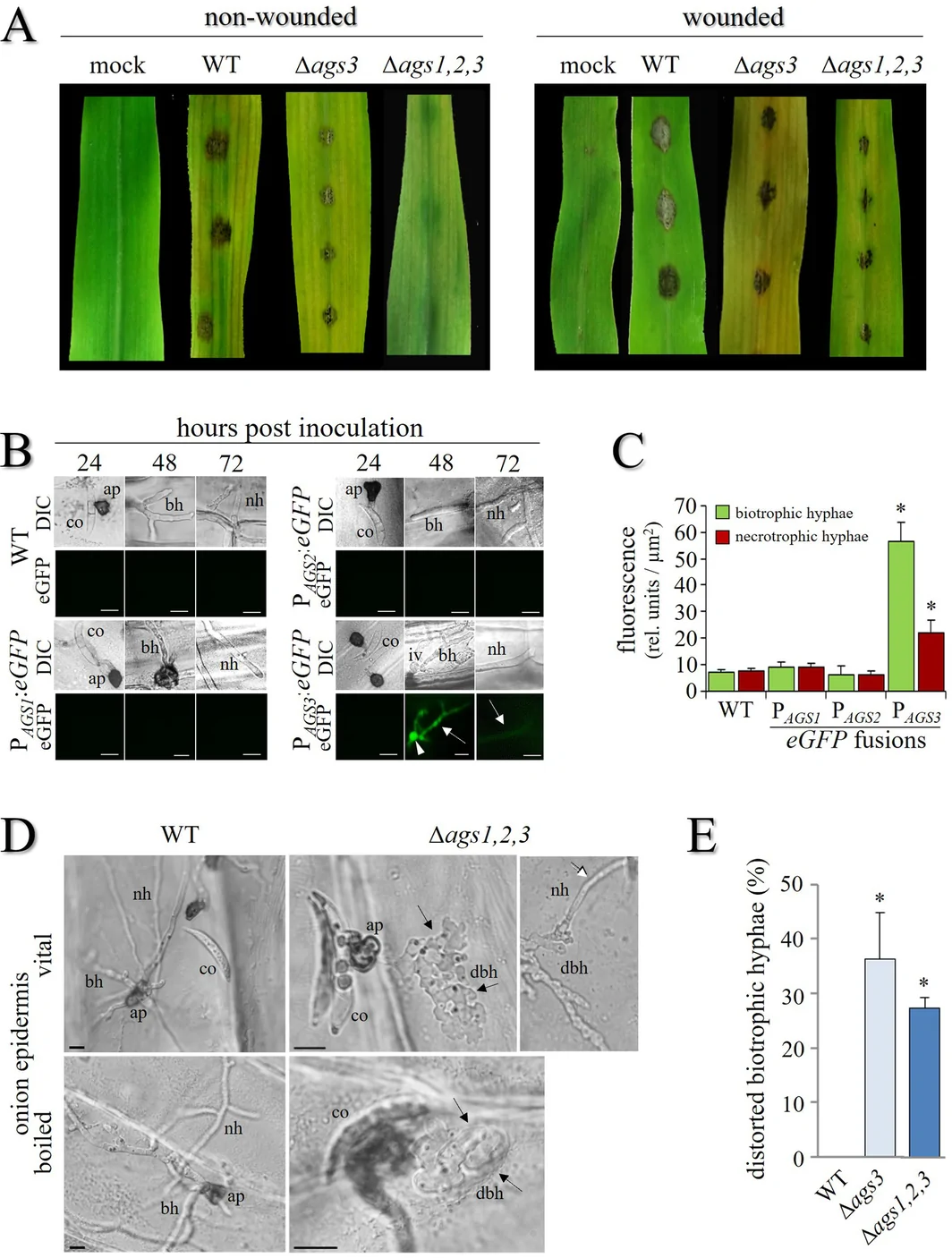
α‐1,3‐glucan is required for full virulence on intact and wounded maize leaves. (A) Infection assays on non‐wounded maize leaf segments revealed strongly reduced virulence of the Δ*ags3* single mutant, and the Δ*ags1,2,3* triple mutant caused no anthracnose disease symptoms. On wounded leaves both Δ*ags3* and Δ*ags1,2,3* mutants caused disease symptoms, but symptom severity caused by these strains was weaker than that caused by the WT. Photos were taken at 3 dpi. (B) Inoculation of maize leaves with transformants harboring promoter‐*eGFP* fusions showed that only *AGS3* was strongly expressed in biotrophic (P_*AGS3*_:*EGFP*, 48 hpi, arrows) and weakly in necrotrophic infection structures (P_*AGS3*_:*EGFP*, 72 hpi, arrow). (C) Quantification of eGFP fluorescence in biotrophic and necrotrophic hyphae of promoter‐*eGFP* strains. eGFP fluorescence of 25 biotrophic and necrotrophic hyphae of transformants expressing P_*AGS1*_:*EGFP*, P_*AGS2*_:*EGFP or* P_*AGS3*_:*EGFP* were quantified. (D) Infection structures differentiated on vital or boiled onion epidermis layers. Conidia (co) of the WT strain differentiated appressoria (ap), which invaded the host and differentiated biotrophic (bh) and necrotrophic hyphae (nh). Comparably, appressoria (ap) of the Δ*ags1,2,3* mutant invaded the epidermis, but formed severely distorted biotrophic hyphae (dbh) in both living, as well as in boiled onion epidermis layers. Cell walls were barely visible (see biotrophic WT hyphae for comparison). Occasionally, phenotypically normal necrotrophic hyphae grew out of distorted biotrophic hyphae (Δ*ags1,2,3*, right panel, nh, arrow), indicating viability of distorted hyphae. (E) Quantification of distorted biotrophic hyphae in WT, Δ*ags3* and Δ*ags1,2,3* mutants. 3 × 100 biotrophic hyphae of each strain were counted. Size bars in (B, D) represent 10 μm. Error bars in (C, E) show standard deviations, asterisks indicate statistical distinctness from the WT at *p* < 0.05.

### 2.6 α‐1,3‐Glucan of *C. graminicola* is Not Required for Evasion of Defense Responses in Maize Leaves

If surface‐masking by α‐1,3‐glucan would interfere with innate immunity in plants and facilitate fungal stealth infection (Fujikawa et al. 2012), one would expect strongly increased transcript abundances of PAMP‐triggered genes when α‐1,3‐glucan‐deficient mutants invade the host plant and form biotrophic hyphae upon direct contact with pattern recognition‐receptors localized in the host plasma membrane (de Oliveira Silva et al. 2022; Gust et al. 2017). At 36 hpi, *C. graminicola* had invaded the host epidermal cell and young biotrophic infection vesicles had begun to invaginate the host plasma membrane. At 48 hpi, voluminous biotrophic primary hyphae had formed (Figure 7B). Therefore, samples of maize leaves inoculated with the WT strain or with the Δ*ags1,2,3* mutant were taken at these time points to analyze host defense responses. We quantified the transcript abundances of previously described PAMP‐response genes, included two extracellular peroxidase genes, one gene each encoding a chitinase or a xyloglucan endotransglucosylase/hydrolase, and two genes encoding terpene synthases (Oliveira‐Garcia and Deising 2013, 2016a). The xyloglucan endotransglucosylase/hydrolase (GRMZM2G392125), which had previously been described as a β‐1,3‐glucanase, showed a strong transcriptional response to *C. graminicola* transformants overexpressing β‐glucan biosynthesis genes (Oliveira‐Garcia and Deising 2013, 2016a), and can therefore be regarded as a PAMP response gene. A triple ectopic strain, harboring all three ectopically integrated *AGS* deletion constructs, served as control (Figure 8). Significant differences in the transcriptional response of the selected PAMP‐responsive genes were not found in leaves inoculated with the α‐1,3‐glucan‐deficient Δ*ags1,2,3* triple mutant at any of the two time points investigated, except for one chitinase gene, which showed weakly but yet significantly increased (*p* < 0.05) transcript abundance at 48 hpi, (Figure 8). These data clearly argue against a role of surface‐localized α‐1,3‐glucan in evasion of PAMP‐triggered immunity.

**FIGURE 8:**
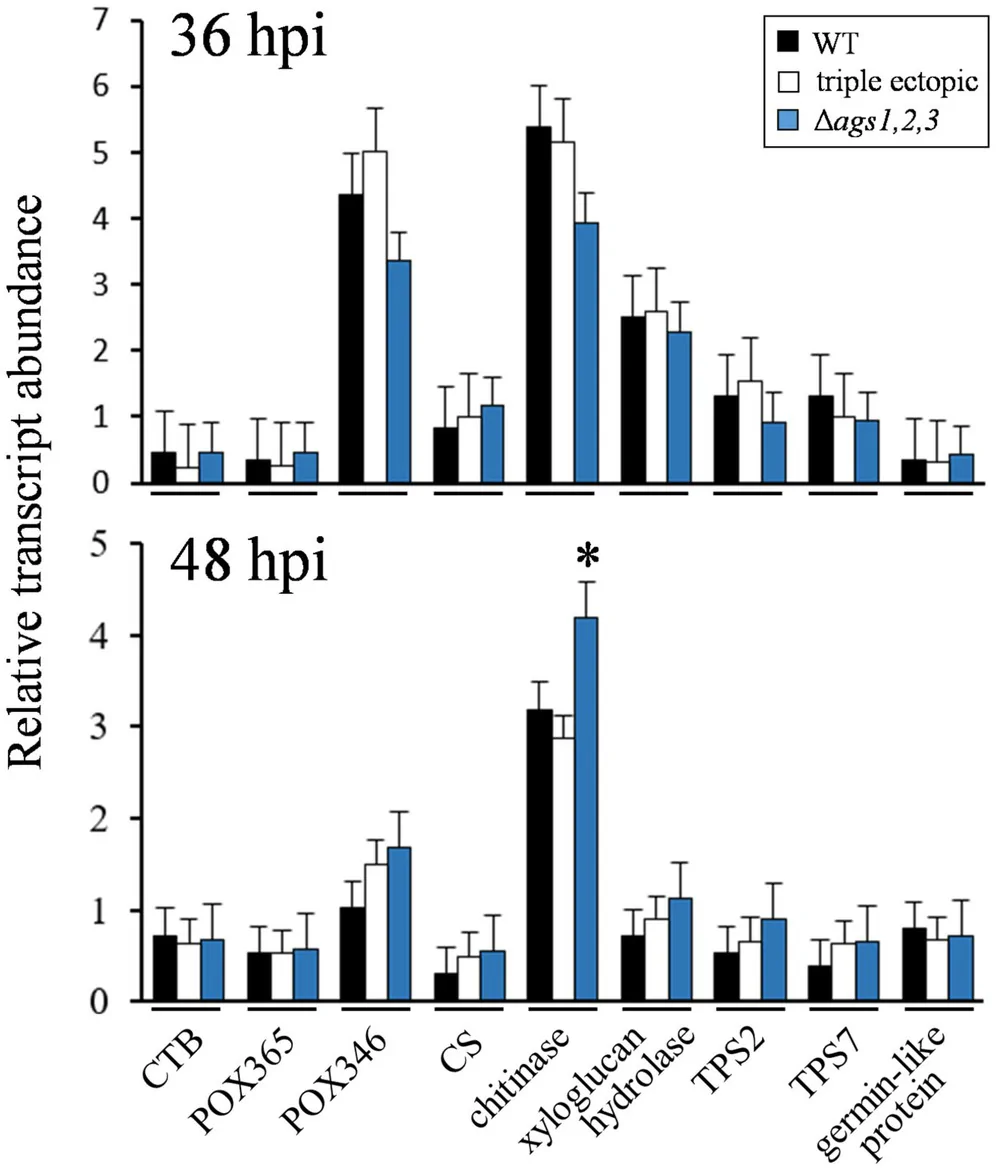
Transcript abundance of PAMP‐responsive genes in maize leaves inoculated with the *C. graminicola* WT strain, the Δ*ags1,2,3* mutant, or the triple ectopic strain containing ectopic integrations of the deletion constructs (control). With the exception of one chitinase gene (48 hpi), no increased transcript abundances were observed in leaves inoculated with the Δ*ags1,2,3* mutant. Error bars represent standard deviations of three biological replicates; asterisk indicates statistical significance at *p* < 0.05.

## 3 Discussion

Early work has suggested a role of α‐1,3‐glucan in cell wall function and virulence of the dimorphic human pathogens *Paracoccidioides brasiliensis, Blastomyces dermatitidis* and *Histoplasma capsulatum* (Hogan and Klein 1994; San‐Blas et al. 1977; Klimpel and Goldman 1988). Experimental evidence that in *H. capsulatum* α‐1,3‐glucan serves as a PAMP‐concealing polymer originated from the finding that α‐1,3‐glucan‐specific antibodies labelled the outer cell wall layer of the pathogenic yeast form of the WT strain, but not that of the *ags1*(Δ) mutant. Moreover, *ags1*(Δ) mutant cells, but not those of the WT yeast, bound to 3 T3 fibroblasts expressing the murine β‐glucan receptor *dectin‐1* (Rappleye et al. 2007). These studies, and others (Marion et al. 2006), have shown that α‐1,3‐glucan of *H. capsulatum* is required for evasion of host defense responses, yeast survival and pathogenicity. However, the cell wall structure and composition of yeasts and filamentous fungi are fundamentally distinct (Gow et al. 2017). Therefore, it was not surprising that mutants of *C. graminicola* lacking all three *AGS* genes and, hence, cell wall α‐1,3‐glucan, did not induce major PAMP‐response genes in the host of this fungus, i.e., *Zea mays* , as shown here. Comparably, changes in transcript abundances of defense genes in rice inoculated with Δ*ags1* mutants of *M. oryzae* were increased approximately 3‐ to 5‐fold, when compared with plants inoculated with the WT (Fujikawa et al. 2012). To illustrate the degree to which PAMP responses can reach, Oliveira‐Garcia and Deising (2013; 2016) have observed several hundred‐ to thousand‐fold increases in defense transcript abundance in maize leaves inoculated with *C. graminicola* mutants overexpressing the β‐glucan biosynthesis genes *GLS1*, *KRE5*, or *KRE6* (Oliveira‐Garcia and Deising 2013, 2016a). As the responses to *C. graminicola* and *M. oryzae* mutants lacking cell wall α‐1,3‐glucan were rather moderate in the respective hosts, it is legitimate to question whether this polymer acts to shield effectively PAMP‐active cell wall polymers, such as β‐glucan or chitin, and suggests further that other mechanisms of PAMP‐masking may be active in filamentous plant pathogenic fungi.

Indeed, in several filamentous fungi, extracellular cell wall‐binding effectors play essential roles in masking of PAMPs (Tanaka and Kahmann 2021; de Jonge and Thomma 2009). Hemibiotrophic fungi such as *Colletotrichum* and *Magnaporthe* species are ideally suited to study hyphal masking, as these species differentiate biotrophic hyphae in direct contact with the PAMP receptor‐harboring plant plasma membrane upon host invasion. Subsequently formed necrotrophic hyphae kill their host cells and therefore do not have to conceal their surfaces (Oliveira‐Garcia and Deising 2013, 2016a; O'Connell et al. 2012). In the Arabidopsis pathogen *Colletotrichum higginsianum*, a large repertoire of genes encoding proteins with chitin‐binding CBM50 modules, known as LysM domains, exist and these have been predicted to act in chitin‐masking (O'Connell et al. 2012; Takahara et al. 2016). Two of these, i.e., ChELP1 and ChELP2, are transcriptionally activated at initiation of the biotrophic phase of infection, and biotrophic hyphae were densely labelled by a monoclonal antibody raised against ChELP2. Electron microscopy of cross sections of biotrophic hyphae revealed labelling across the cell wall by a chitin‐binding probe, i.e., wheat germ agglutinin (WGA), suggesting that the LysM effector specifically shielded the surface of biotrophic infection structures. By contrast, chitin was fully accessible to the WGA probe on the surface of necrotrophic hyphae. Both chitin‐binding LysM effectors of *C. higginsianum* are indispensable for suppressing chitin‐triggered immunity and for full virulence on Arabidopsis (Takahara et al. 2016). Moreover, the secreted LysM protein 1 (Slp1) is enriched on the surface of biotrophic hyphae of *M. oryzae* , and is a pre‐requisite for suppression of chitin‐triggered immunity and initiation of rice blast disease (Mentlak et al. 2012). If surface‐localized α‐1,3‐glucan would indeed cover and shield the surfaces of pathogenic hyphae and interfere with PAMP recognition, as suggested by Fujikawa et al. (2012), deletion, or RNAi‐mediated reduction of transcript abundances, of LysM genes would likely not have resulted in massive stimulation of plant‐defense responses and fungal avirulence (Takahara et al. 2016; Mentlak et al. 2012).

Thus, the facts that (i) α‐1,3‐glucan is present across the cell wall and not primarily located on the surface of the hyphal cell wall, (ii) defense responses in maize and rice leaves were only marginally induced by α‐1,3‐glucan‐deficient mutants, and (iii) LysM effectors can physically access chitin on the surfaces of biotrophic hyphae and prevent activation of PAMP‐triggered immunity (Takahara et al. 2016; Mentlak et al. 2012) strongly argue against the applicability of the concept established for *H. capsulatum* to filamentous plant pathogenic ascomycetes and suggest that surface‐localized α‐1,3‐glucan of filamentous fungi is not required for PAMP‐masking and attenuation of plant innate immunity.

Instead, the cell wall permeability experiments shown here indicate that α‐1,3‐glucan reduces cell wall pore diameters and likely restricts the mobility of cell wall‐modifying enzymes such as chitinases, *endo*‐cleaving β‐glucanases or β‐1,3‐glucanosyltransferases, which exhibit molecular masses of 20–60 kDa (Hartl et al. 2011; Caseiro et al. 2022; Hartland et al. 1996; Samalova et al. 2017; Mouyna et al. 2013). Permeability experiments with fluorescein‐dextran‐conjugates of different sizes have shown that in contrast to *C. graminicola* WT cell walls, cell walls devoid of α‐1,3‐glucan permit increased mobility to molecules of up to 70 kDa, suggesting increased accessibility of substrates to fungus‐encoded cell wall‐modulating enzymes, increased cleavage of glycosidic bonds and, hence, softening of structural cell wall polymers in these mutants. Cell wall‐softening is a pre‐requisite for hyphal branching rates (Adams 2004; Harris 2019; Mouyna et al. 2013; Verdín et al. 2019), and, consistently, the eGFP‐tagged chitinase ChiA of *Aspergillus nidulans* localized to hyphal branching sites (Yamazaki et al. 2008). Increased numbers of conidial germ tubes and of hyphal branching may thus indirectly indicate cell wall‐softening in α‐1,3‐glucan‐defective mutants of *C. graminicola* . Interestingly, mutants of *A. fumigatus* lacking all three *AGS* genes showed reduced retention of cell wall proteins, as indicated by a complex pattern of proteins in a NaCl wash of mutant conidia. Conidia of the parental strain, in contrast, did not release any proteins detectable by silver‐staining of SDS‐gels, and proteome analyses identified thirty‐four proteins released by the *ags*Δ mutant, most of which were cell‐wall hydrolases (Beauvais et al. 2013). These data strongly support the hypothesis for a role of α‐1,3‐glucan in reducing protein mobility in fungal cell walls.

Increased mobility of cell wall‐modifying enzymes may also explain lysis of infection structures of α‐1,3‐glucan‐deficient mutants of *M. oryzae* formed on rice cuticles, i.e., before invading the plant (Fujikawa et al. 2012). As chitinases and β‐glucanases are not predominantly present on, or absent from, plant cuticles, it is likely that lysis of appressoria and loss of pathogenicity were probably due to autolysis. On artificial surfaces, lysis of infection structures of α‐1,3‐glucan‐deficient mutants of *M. oryzae* did not occur (Fujikawa et al. 2012), but these experiments do not strengthen the idea that host enzymes caused appressorial disintegration *on planta*. Genes encoding cell wall‐modifying enzymes may be differentially expressed, depending on the stimulus used to induce appressorium formation. Indeed, genome‐wide comparison of gene expression profiles during appressorium formation of *M. oryzae* either induced chemically by cAMP, or by direct inoculation of conidia onto a hydrophobic substratum revealed 357 distinctly regulated genes, including a β‐1,3‐glucanase and three chitinase genes (Oh et al. 2008). As in *C. graminicola* , cell wall pores in *M. oryzae* and the mobility of cell wall‐modifying enzymes such as β‐1,3‐glucanases and three chitinases may be significantly increased in α‐1,3‐glucan‐deficient mutants and could account for cell wall destruction.

In contrast to the rice blast fungus, α‐1,3‐glucan‐deficient mutants of *C. graminicola* differentiate phenotypically normal appressoria. However, some of these infection cells grew out laterally to form a secondary appressorium, which is indicative of softening of appressorial cell walls. Yet, several appressoria of the mutants lacking *AGS3* invaded the maize epidermis directly to form biotrophic hyphae. Transformants harboring *AGS* promoter‐*eGFP* fusions showed strong expression of *AGS3* in biotrophic hyphae, suggesting the requirement for α‐1,3‐glucan particularly for these cells. Correspondingly, biotrophic hyphae of *AGS3*‐deficient mutants differentiated in both living and heat‐inactivated onion cells were severely distorted, strengthening the view that host defense enzymes are not likely to be causal for fungal cell wall lysis.

A further argument against a role of α‐1,3‐glucan in PAMP‐masking in *C. graminicola* is the demonstration that expression of key β‐1,3‐glucan biosynthesis genes, i.e., *GLS1*, *KRE5*, and *KRE6*, and synthesis of β‐1,3‐glucan are massively down‐regulated during biotrophic development, which correlates with pronounced expression of *AGS3* (Oliveira‐Garcia and Deising 2013, 2016a). If α‐1,3‐glucan would play a role in masking of PAMPs on the surface of biotrophic hyphae, strong reduction of β‐1,3‐glucan biosynthesis would not be required and induction of PAMP‐responsive genes would not have remained largely unaltered in leaves inoculated with the Δ*ags1,2,3* mutant, in comparison with leaves inoculated with the WT strain. Recent solid‐state NMR studies have shown that in an *A. fumigatus* WT strain only three cell wall polymers, namely chitin, α‐1,3‐ and β‐1,3‐glucan are responsible for hyphal rigidity, with strong interactions between chitin and α‐1,3‐glucan resulting in formation of a hydrophobic core structure (Kang et al. 2018). Moreover, solid‐state NMR analyses of α‐1,3‐glucan‐deficient mutants of this fungus revealed that in the absence of α‐1,3‐glucan, chitin polymer chains are tightly packed and show enhanced rigidity, and that the structural roles of chitin and α‐1,3‐glucan are not interchangeable (Chakraborty et al. 2021). If the *A. fumigatus* cell wall model (Kang et al. 2018; Chakraborty et al. 2021) would be applicable to the cell wall architecture of *C. graminicola* , down‐regulation of β‐1,3‐glucan biosynthesis (Oliveira‐Garcia and Deising 2013, 2016a) would leave chitin remaining as the only structural polymer in biotrophic hyphae of α‐1,3‐glucan‐deficient mutants, and, as discussed above, cell wall architecture in these mutants may be further compromised by increased mobility of cell wall‐modifying enzymes. Interestingly, formation of phenotypically normal necrotrophic hyphae from distorted biotrophic hyphae of α‐1,3‐glucan‐deficient mutants of *C. graminicola* suggests that re‐constitution of β‐1,3‐glucan biosynthesis in necrotrophic hyphae (Oliveira‐Garcia and Deising 2013, 2016a) and the presence of chitin are sufficient for the development of these structures. This is in agreement with reduced expression of *AGS3* in necrotrophic hyphae.

Collectively, our data suggest that in *C. graminicola* α‐1,3‐glucan does not play a role in PAMP‐masking and attenuation of plant innate immunity. Instead, our results are consistent with roles of this cell wall polymer in controlling molecular mobility within the cell wall and cell wall architecture, which may explain the observed reduction in virulence of this hemibiotrophic fungal maize pathogen.

## 4 Experimental Procedures

### 4.1 Strains, Media, Culture Conditions and Growth Assays

The wild‐type (WT) strain CgM2 of *C. graminicola* (Ces.) G.W. Wilson (teleomorph *Glomerella graminicola* D.J. Politis) and mutant strains generated in this study were cultivated on oat meal agar (OMA; Werner et al. 2007), potato‐dextrose (PD; Difco Laboratories, Sparks, MD, USA), complete medium (CM; 10 g glucose, 1 g yeast extract, and 1 g casein hydrolysate (Difco Laboratories, Augsburg, Germany) in double‐distilled water, 10 mL of a stock solution containing 50 g Ca(NO_3_)_2_ per 500 mL, and 10 mL of a stock solution containing 10 g KH_2_PO_3_, 12.5 g MgSO_4_·7H_2_O, and 2.7 g NaCl per 500 mL). The final volume was adjusted to 1 L with double‐distilled water. Alternatively, minimal medium (MM; 10 g glucose; 1 g Ca(NO_3_)_2_; 0.2 g KH_2_PO_3_; 0.25 g MgSO_4_ and 0.054 g NaCl per 1 L of re‐distilled water) was used. Regeneration medium contained 342.3 g sucrose, 1 g yeast extract, 1 g casein and 6 g agar‐agar (all from Difco Laboratories, Sparks, MD, USA) in 1 L of double‐distilled H_2_O.

For growth in liquid media, strains were kept in an incubation shaker (Unitron, Infors, Bottmingen, Switzerland) at 110 rpm and 23°C. For growth on solidified media, containing 1.5% (w/v) agar‐agar (Difco Laboratories, Sparks, MD, USA), strains were kept at 23°C under continuous fluorescent light (Climas Control CIR, UniEquip, Martiensried, Germany; Werner et al. 2007).

To challenge cell wall biosynthesis, strains were grown on PDA (Becton Dickinson GmbH, Heidelberg, Germany) supplemented with the cell wall‐disturbing agents Caspofungin (0.032 μg/mL; Hexal AG, Holzkirchen, Germany; Serfling et al. 2007) or Nikkomycin Z (24.7 μg/mL; Sigma‐Aldrich, Taufkirchen, Germany; Werner et al. 2007) at 23°C.

### 4.2 Infection Assays

Fourteen‐day‐old *Zea mays* cv. Mikado plants (KWS SAAT AG, Einbeck, Germany) were grown and used in infection assays as described (Oliveira‐Garcia and Deising 2013). Briefly, segments (8 to 10 cm) of the second and third fully developed intact leaf were inoculated with 10 μL droplets containing 10^6^ conidia per ml in 0.01% (vol/vol) Tween 20. For wound‐inoculation assays, maize leaves were wounded by inserting five pricks using a sterile lancet (DB Micro‐Fine, 0.3 mm, 30G, Becton Dickinson, Franklin Lakes, NJ, USA) immediately before inoculation. For microscopy, inoculation was done using 10 μL droplets containing 10^5^ conidia per ml in 0.01% (vol/vol) Tween 20. Leaf segments were placed in sealed petri dishes (diameter 9 cm), and photographs were taken at 4 dpi (Nikon D50, Nikon, Düsseldorf, Germany).

For simple visualization of infection structures, infection assays were performed using single layers of onion ( *Allium cepa* cv. Grano) epidermis cells as an alternative host. Adaxial epidermal layers were positioned onto sterile water agar (0.7% (w/v)) and inoculated with 10 μL droplets containing 10^5^ conidia per ml in 0.01% (vol/vol) Tween 20. Heat‐inactivation of enzymes was done by incubating epidermal cell layers in tap water at 95°C for 15 min.

### 4.3 Targeted Mutagenesis of *AGS* Genes and Construction of *AGS* : *eGFP* Strains

The 5′‐and 3′‐flanking regions used to assemble the deletion construct of *AGS1* of 1000 bp each were PCR‐amplified from genomic DNA using primers AGS1‐P‐fw and AGS1‐P‐hyg.rev, and AGS1‐T‐hyg.fw and AGS1‐T‐rev, respectively. The flanks of the *AGS2*‐deletion constructs, which were approx. 600 bp, were amplified using primer pairs AGS2‐P‐fw and AGS2‐P‐nours.rev, and AGS2‐T‐nours.fw and AGS2‐T‐rev, respectively. The 5′‐and 3′‐flanks of the *AGS3*‐deletion construct of approx. 800 bp each were amplified using primer pairs AGS3‐P‐fw and AGS3‐P‐G418.rev, and AGS3‐T‐G418.fw and AGS3‐T‐rev. The *hph*, *nat1*, and *nptII* resistance cassettes were PCR‐amplified from plasmids pAN7‐1 (Punt et al. 1987), pNRl (Malonek et al. 2004), and pII99 (Beck et al. 1982), using primer pairs uni‐hyg.F1 and uni‐hyg.R1, uni‐nours.F1 and uni‐nours.R1, and uni‐G418.F1 and uni‐G418.R1, respectively. The amplified 5′‐and 3′‐flanks of *AGS1*, *AGS2*, and *AGS3* were ligated with the *hph*, *nat1*, and *nptII* resistance cassettes using double‐joint‐PCR (DJ‐PCR; Yu et al. 2004), and the products were blunt‐end‐ligated into pJET1.2/*blunt*, using the CloneJET PCR Cloning Kit (Fermentas, St. Leon‐Rot, Germany) and *Phusion*‐High Fidelity DNA Polymerase (FinnZymes, Espoo, Finland). Plasmids containing the deletion constructs were transformed into *E. coli‐*XL1‐Blue cells (Chung et al. 1989). Deletion constructs for *AGS1*, *AGS2*, and *AGS3* were amplified from plasmids using the corresponding nested primer pairs AGS1‐P‐fw.nest and AGS1‐T‐rev.nest, AGS2‐P‐fw.nest and AGS2‐T‐rev.nest, and AGS3‐P‐fw.nest and AGS3‐T‐rev.nest, respectively.

In order to generate double deletion mutants Δ*ags1,2* and Δ*ags1,3*, *AGS2* and *AGS3* deletion constructs were transformed into Δ*ags1* strains. Transformation of the *AGS3* deletion construct into Δ*ags1,2* strains yielded the triple deletion mutant Δ*ags1,2,3*. Integration by homologous recombination was confirmed by PCR and genomic Southern blot analyses.

The *AGS*‐promoter:*eGFP* constructs, i.e., P_*AGS1*_:*eGFP*, P_*AGS2*_:*eGFP*, and P_*AGS3*_:*eGFP* consisted of the 1.380 bp, 819 bp, and 1.098 bp upstream of the start codon, of *AGS1*, *AGS2*, and *AGS3*, respectively, the 1.111 bp *eGFP* fragment including the T*nos* terminator, the nourseothricin‐resistance cassette consisting of the *oliC* promoter, the *nat1* gene, and the *Botrytis cinerea tub1* terminator (Malonek et al. 2004). The promoter regions were PCR‐amplified from WT genomic DNA using primer pairs PAGS1_Fw and PAGS1_Rv; PAGS2_Fw and PAGS2_Rv; and PAGS3_Fw and PAGS3_Rv. The entire nourseothricin‐resistance cassette was PCR‐amplified from plasmid pNR1 (Malonek et al. 2004), using primers uni‐nours.F1 and uni‐nours.R1 (Table S1).

P_*AGS1*_‐, P_*AGS2*_‐ and P_*AGS3*_‐fragments were fused with the *eGFP* fragment, including the T*nos* terminator, and the nourseothricin‐resistance cassette by DJ‐PCR (Yu et al. 2004). The products were directly used in a second PCR reaction using nested primers AGS1‐P‐fw.nest and AGS1‐P‐rev.nest, AGS2‐P‐fw.nest and AGS2‐P‐rev.nest, and AGS3‐P‐fw.nest and AGS3‐P‐rev.nest, respectively. The final amplified promoter:*eGFP* constructs had sizes of 4.647 bp (P_*AGS1*_:*eGFP*), 4.079 bp (P_*AGS2*_:*eGFP*), and 4.178 bp (P_*AGS3*_:*eGFP*) and were directly transformed into protoplasts generated from oval conidia. Putative transformants were selected on plates containing 100 μg/mL nourseothricin, and colonies containing the *eGFP* constructs were identified by genomic Southern blot experiments (see below).

### 4.4 DNA and RNA Extraction and Genomic Southern Blots

Extraction of genomic DNA and Southern blot analyses were performed as described (Werner et al. 2007). To analyze transformants for correct deletion of *AGS1*, *AGS2*, and *AGS3* and for the number of integrations of deletion or *eGFP* constructs, 10 mg of DNA digested with the appropriate restriction endonuclease was used. Alkali‐labile DIG‐dUTP‐labeled probes (Roche Diagnostics) specific for the nourseothricin acetyl transferase gene *Nat‐1*, the hygromycin phosphotransferase gene *hph*, and the geneticin resistance gene neomycin phosphotransferase II were amplified from plasmid pNRl (Malonek et al. 2004) using primers NatR‐probe‐Fw and NatR‐probe‐Rv, pAN7‐1 (Punt et al. 1987), using primers Hyg‐probe‐Fw and Hyg‐probe‐Rv, and from pII99 (Beck et al. 1982), using primers G418‐probe‐Fw and G418‐probe‐Rv (Table S1).

### 4.5 RT‐qPCR

qRT‐PCR experiments were performed in a total volume of 20 μL, using 100 ng of total RNA pretreated with RQ1 RNase‐free DNase (Promega) and the Power SYBR Green RNA‐to‐CT 1‐step kit (Applied Biosystems), following the instructions of the manufacturer. Primers used are shown in Table S1. Melting curve and agarose gel analyses were done to confirm the amplification of a single product. Transcript abundance was calculated and normalized as described (Gutjahr et al. 2008), using the histone H2B and the β‐tubulin transcripts of maize. Cycle threshold values of three independent replicates were used to calculate mean values and standard deviations. Normalized transcript abundances are shown relative to the abundances in non‐inoculated leaves.

### 4.6 Chemical Cell Walls Analysis

*Colletotrichum graminicola* wild type and the Δ*ags1,2,3* mutant were grown on OMA for 7 days at 28°C. Conidia were suspended in deionized water, and 2 × 10^5^ conidia were inoculated into 150 mL potato dextrose broth and incubated for 7 to 12 days at 28°C and 80 rpm. The mycelium was filtered through Miracloth (Merck, Darmstadt, Germany), washed with ultrapure water, filtered again through Miracloth (Merck, Darmstadt, Germany) and snap frozen in liquid nitrogen. Subsequently, the mycelium was ground in liquid nitrogen and extracted with 70% (v/v) ethanol overnight under rotation, and twice for 1 h at 90°C, to obtain the alcohol‐insoluble residue (AIR). Proteins were extracted by incubating the AIR three times with 50 mM Tris–HCl buffer (pH 7.8) containing 2% (w/v) sodium dodecyl sulfate (SDS), 40 mM 2‐mercaptoethanol, and 10 mM EDTA as described (Mélida et al. 2013). After washing three times with ultrapure water, the purified cell walls were lyophilized and subjected to alkali‐fractionation as described by Mélida et al. (2013). Briefly, cell wall material was extracted three times with 80% (v/v) methanol containing 5% (w/v) KOH and 0.1% (w/v) NaBH_4_ at 100°C for 15 min. The alkali‐insoluble fraction (AIF) was collected via centrifugation and washed once with 0.5 M acetic acid and three times with ultrapure water. The first wash was added to the alkali‐soluble fraction (ASF), which was subsequently adjusted to pH 6 with acetic acid and dialyzed against ultrapure water through 3.5 kD MWCO Spectra/Por3 membranes (Spectrum Labs, San Francisco, CA, USA). Lyophilized AIR, AIF and ASF were subjected to cell wall hydrolysis. Materials were incubated with 72% (w/w) sulfuric acid for 1 h at RT and vortexed every 15 min. Samples were then diluted to 4% (w/v) sulfuric acid and hydrolyzed by autoclaving at 121°C for 60 min. After removal of insoluble material by centrifugation, hydrolysates were diluted 30‐fold in ultrapure water. 50 μM ribose was added as an internal standard and monosaccharides were quantified via high‐performance anion‐exchange chromatography with pulsed amperometric detection (HPAEC‐PAD) as described (Reckleben et al. 2025). Monosaccharides were separated on a biocompatible Knauer Azura HPLC system (Eckert & Ziegler Eutrope GmbH, Berlin, Germany) equipped with Thermo Fisher Dionex CarboPac PA20 BioLC analytical (3 × 150 mm) and guard (3 × 30 mm) columns (Thermo Fisher, Waltham, MA, USA) in a solvent gradient of (B) 10 mM NaOH and (C) 700 mM NaOH in ultrapure water at 0.4 mL/min flow rate: 0 to 25 min: 20% B; 25 to 28 min: 20% to 0% B, 0% to 70% C; 28 to 33 min: 70% C; 33 to 35 min: 70% to 100% C; 35 to 38 min: 100% C; 38 to 42 min: 0% to 20% B, 100% to 0% C; 42 to 60 min: 20% B. Monosaccharides were detected via an Antec Decade Elite SenCell (Boston, MA, USA) heated to 40°C and calibrated against pure monosaccharide standards (Sigma‐Aldrich, Taufkirchen, Germany).

### 4.7 Analysis of Protein Release From *C. graminicola* AIR

For profiling protein release from *C. graminicola* AIR preparations, 15 mg AIR (paragraph 4.6) was incubated in protein extraction buffer (50 mM Tris–HCl pH 7.8, 2% (w/v) SDS, 40 mM 2‐mercaptoethanol, 10 mM EDTA) for 10 min at 80°C. The protein concentration was determined using the Bradford assay (Serva, Heidelberg, Germany), and 37.5 μg of total protein per sample was loaded onto a 12% SDS‐PAGE gel. After separation, the gel was stained with Coomassie Brilliant Blue R‐250, and bands of an M_r_ range of approx. 20 kDa and 130 kDa were excised for proteomic analysis.

After‐destaining, samples were reduced, carbamidomethylated, and digested “in‐gel” by the addition of Sequencing Grade Modified Trypsin (Serva, Heidelberg, Germany) and incubated at 37°C overnight. Peptides were desalted and concentrated using Chromabond C18WP spin columns (Macherey‐Nagel, Düren, Germany). Finally, peptides were dissolved in 25 μL of water with 5% acetonitrile and 0.1% formic acid. The mass spectrometric analysis of the samples was performed using an Orbitrap Velos Pro mass spectrometer (Thermo Scientific, Waltham, MA, USA). An Ultimate nanoRSLC‐HPLC system (Dionex, Germering, Germany), equipped with a custom end‐fritted 50 cm x 75 μm C18 RP column filled with 2.4 μm beads (Dr. Maisch HPLC, Ammerbuch‐Entringen, Germany), was connected online to the mass spectrometer through a Proxeon nanospray (Proxeon, Odense, Denmark) source. Subsequently, 6 μL of the tryptic digest were injected onto a 300 μm ID × 1 cm C18 PepMap pre‐concentration column (Thermo Scientific, Waltham, MA, USA). Automated trapping and desalting of the sample were performed at a flow rate of 6 μL/min using water/0.05% formic acid as solvent. Separation of the tryptic peptides was achieved with the following gradient of water/0.05% formic acid (solvent A) and 80% acetonitrile/0.045% formic acid (solvent B) at a flow rate of 300 nL/min: holding 4% B for 5 min, followed by a linear gradient to 45% B within 30 min and linear increase to 95% solvent B in an additional 5 min. The column was connected to a stainless steel nanoemitter (Proxeon, Odense, Denmark), and the eluent was sprayed directly towards the heated capillary of the mass spectrometer using a potential of 2300 V. A survey scan with a resolution of 60,000 within the Orbitrap mass analyzer was combined with at least three data‐dependent MS/MS scans with dynamic exclusion for 30 s either using CID with the linear ion‐trap or using HCD combined with orbitrap detection at a resolution of 7500. Data analysis was performed using Proteome Discoverer 2.2 (Thermo Scientific, Waltham, MA, USA) with SEQUEST search engine.

### 4.8 Environmental Scanning Electron Microscopy (ESEM)

ESEM studies were performed after inoculating the *C. graminicola* WT and Δ*ags1,2,3* triple deletion mutant onto Poly‐L‐Lysine‐coated microscopy slides (LabScientific, Danvers, USA). Conidia were suspended in complete medium containing 2% (w/v) sucrose to stimulate formation of vegetative hyphae. For appressorium differentiation, sucrose was omitted.

ESEM was done without sample drying or coating, using an ESEM (Quanta3D FEG Dual‐Beam, FEI Company, Hillsboro, OR, USA) in a water vapor atmosphere, as described (Küster et al. 2008).

### 4.9 Atomic Force Microscopy (AFM) and Measurement of Young's Module

Atomic force microscopy (AFM)‐based nanoindentation (NanowizardII, JPK Instruments, Berlin, Germany) was used for recording topographical information as well as local mechanical properties of appressorial surfaces. Glass slides were placed in petri dishes and sprayed with 10 mL of a suspension containing 10^6^ spores/mL. Spores were allowed to settle for 1 h, and non‐adhering spores were removed by washing with sterile distilled H_2_O. Petri dishes containing the glass slides were incubated at 23°C in darkness for 48 h. AFM was applied in the force‐mapping mode to an area of 2 × 2 μm, yielding 128 × 128, i.e., 16,384 measurement points. All measurements were performed in a standard liquid cell (JPK Instruments, Berlin, Germany) in distilled water. Young's moduli were calculated from the load penetration curve according to an advanced Hertzian model for spherical indenter geometry (Johnson 1987), using post processing software JPK‐Data processing (JPK Instruments, Berlin, Germany).

### 4.10 Light Microscopy

Bright Field (BF), Differential Interference Contrast (DIC), and fluorescence microscopy were done using an Eclipse E600 microscope. Micrographs were taken using a Digital Slight DS‐U3 camera.

Hyphal branching rates were quantified using the NIS‐Elements Advanced Research Version 3.00 (Nikon, Düsseldorf, Germany), with 100 branching distances measured in three biological replicates of the WT and Δ*ags3* strains.

To detect eGFP fluorescence, a super high‐pressure Mercury lamp and a UV‐2A 420 filter block (Nikon, Düsseldorf, Germany) were used. Staining with MOPC 104E was done as follows: Hyphae that had developed on poly‐L‐lysine‐coated microscopy coverslips (Sigma‐Aldrich, Saint Louis, MO, USA) were washed once with 1× phosphate‐buffered saline (PBS) for 5 min. Non‐specific binding was blocked by incubating the samples with 1% (w/v) bovine serum albumin (BSA) in PBS (150 μL per slide) for 30 min at room temperature. The primary mouse IgM antibody MOPC‐104E (Sigma‐Aldrich, Saint Louis, MO, USA) was then applied, and slides were covered with plastic strips and incubated for 2 h at 30°C in a humid chamber. After primary antibody incubation, slides were washed three times with PBS for 10 min each. The Alexa Fluor 488‐conjugated secondary antibody (5 μg/mL) (Thermo Fisher Scientific, Waltham, MA, USA) was applied and incubated for 1 h at 30°C in a dark, humid chamber. Slides were subsequently washed three times with PBS for 10 min each. Finally, coverslips were mounted onto glass slides using mounting medium (Thermo Fisher Scientific, Waltham, MA, USA), and samples were used for fluorescence microscopy.

Staining with the DS1‐GFP protein (Suyotha et al. 2013) was done as described. Briefly, *E. coli* cells harboring recombinant DS1‐GFP fusions were cultivated in LB medium supplemented with 95 μg/mL ampicillin and incubated at 37°C with shaking until the culture reached an OD_600_ of 0.5 (approximately 2.5 h). Expression of secreted DS1‐GFP was induced by adding IPTG to a final concentration of 0.4 mM, followed by incubation at 25°C overnight. The cell suspension was centrifuged at 4°C, and the supernatant was used directly for hyphal staining (Suyotha et al. 2013). Fungal hyphae were grown in shaking cultures in Complete Medium, collected by centrifugation (4°C; 2000 x *g*; 15 min), washed with 1× PBS and incubated with DS1 for 2 h in the dark. Subsequently, hyphae were washed with PBS and then with distilled water for 5 min, mounted on glass slides and examined by fluorescence microscopy using a GFP filter as specified above.

eGFP‐fluorescence of infection hyphae of WT and strains harboring promoter‐eGFP fusions was quantified using ImageJ software (Jensen 2013).

DIC microscopy was used as described (Oliveira‐Garcia and Deising 2016b).

To visualize fungal structures in leaf tissues, maize leaves were destained in MeOH/acetic acid (3:1) for 48 h and subsequently submerged in 0.1% (v/v) aniline blue for 3 min, washed in water, and analyzed by DIC or BF microscopy (Hood and Shew 1996).

### 4.11 Fluorescence Imaging and FRAP Experiments

Fluorescence imaging experiments were performed on a Zeiss LSM 880 Airyscan system (Carl Zeiss, Thornwood, NY, USA), equipped with a 40× Plan‐Apochromat objective (NA 0.95). Fluorescein isothiocyanate‐labelled dextrans of 4, 20 and 70 kDa (FD4, FD20S, FD70S; Sigma‐Aldrich, Schnelldorf, Germany) were used at a final concentration of 50 μM. Samples were incubated with the dextran solutions for 12 min and subsequently rinsed five times with distilled water. Photobleaching was achieved using a rectangular region of interest illuminated with the 488 nm and 514 nm lines of a 40‐mW argon laser operated at 25% laser power. Fluorescence recovery was monitored at low laser intensity (0.24% of 40 mW) with images acquired at 10 s intervals. Data analysis and curve fitting were performed using the freely available *K_FRAPcalcV9k.ipf* procedure (Kota Miura, CMCI, EMBL Heidelberg, Germany). Individual curves were analyzed to determine the means (and standard deviations) of the recovery times and mobile fractions for each condition.

### 4.12 Immunogold Labelling and Transmission Electron Microscopy

For immunogold labelling and transmission electron microscopy, high pressure freezing followed by cryo‐substitution and resin‐embedding was employed (Table S2), Transversal leaf vibratome sections of 200 μm thickness and a size of 5 × 3 mm were used for High Pressure Freezing (HPF) with a HPF Compact 03 (Engineering Office, M. Wohlwend GmbH, CH‐9466 Sennwald). Sections were transferred into aluminum platelets with 6 mm Ø and deepening of 0.2 mm. A mixture of yeast and cyanobacteria (3:1) was used as filler for the platelets. The flat side of platelets with 0.3 mm deepening was used as lid to close the 0.2 mm platelet. The platelet sandwich was used for HPF at 2000 bar. Freeze substitution in an automated FS unit (Leica Microsystems, Bensheim, Germany), Lowicryl HM20 resin embedding after high pressure freezing and UV‐polymerization was performed as shown in Table S2.

Ultrathin sectioning, immunogold labelling using primary mouse IgM antibody MOPC‐104E (Sigma‐Aldrich, Saint Louis, MO, USA), Goat anti‐Mouse IgG (10 nm Gold), and transmission electron microscopy analysis was performed as described (Demarchi et al. 2023).

### 4.13 Molecular Phylogeny

Multiple sequence alignments were done using ClustalW (http://embnet.vital‐it.ch/software/ClustalW.html; Larkin et al. 2007). Phylogenetic dendograms were constructed using MEGA 5 (www.megasoftware.net; Tamura et al. 2011), with the minimum evolution algorithms using 1000 bootstrap replications. Sequences of all fungal α‐1,3‐glucan synthase genes were obtained from the database of the National Center for Biotechnology Information (http://www.ncbi.nlm.nih.gov/).

### 4.14 Statistics

All experiments, if not otherwise stated, were performed in three biological replicates using the WT strain and at least three independent mutants. If not otherwise noted, calculation and statistical analysis of differences between groups were done using the one‐way ANOVA test, followed by a Tukey–HSD test at the alpha degree (*p* < 0.05) (Microsoft Excel, Microsoft Deutschland GmbH, Unterschleißheim, Germany).

### 4.15 Accession Numbers of α‐1,3‐Glucan Synthase Proteins Used in This Study

**Table:** 

Protein (NCBI‐annotation)	Species	Accession number
AfAgs1	*Aspergillus fumigatus*	XP_748412.1
AfAgs2	*Aspergillus fumigatus*	XP_755460.1
AfAgs3	*Aspergillus fumigatus*	XP_752912.1
CgAgs1	*Colletotrichum graminicola*	EFQ32048.1
CgAgs2	*Colletotrichum graminicola*	EFQ34679.1
CgAgs3	*Colletotrichum graminicola*	EFQ35820.1
ChAgs1	*Colletotrichum higginsianum*	XP_018157918.1
ChAgs2	*Colletotrichum higginsianum*	XP_018159265.1
CnAGS	*Cryptococcus neoformans*	XP_572121.1
MoAGS1	*Magnaporthe oryzae*	XP_003721444.1
PbMok11	*Paracoccidioides brasiliensis Pb03*	EEH18163.1
PbMok13	*Paracoccidioides brasiliensis Pb18*	EEH47071.1
SpAgs1	*Schizosaccharomyces pombe*	NP_588165.3
SpMok11	*Schizosaccharomyces pombe*	NP_001018282.1
SpMok12	*Schizosaccharomyces pombe*	NP_595619.1
SpMok13	*Schizosaccharomyces pombe*	NP_596500.1
SpMok14	*Schizosaccharomyces pombe*	NP_587978.1

## Author Contributions

**Iris Gase:** investigation, validation, supervision, writing – review and editing. **Lala Aliyeva‐Schnorr:** investigation, validation, supervision, writing – review and editing. **Michael Melzer:** investigation, validation, writing – review and editing. **Maria Ott:** investigation, validation, writing – review and editing. **Julia Haufe:** investigation. **Matthias Menzel:** investigation. **Timo Engelsdorf:** investigation, validation, writing – review and editing. **Christiane Rohrbach:** validation, investigation. **Alan de Oliveira Silva:** investigation, software. **Lars M. Voll:** validation, supervision, writing – review and editing. **Alexander S. Mickel:** investigation, validation, writing – review and editing. **Maximilian Groß:** investigation, writing – review and editing. **Mamoru Wakayama:** investigation. **Holger B. Deising:** conceptualization, writing – original draft, writing – review and editing, supervision.

## Ethics Statement

The authors have nothing to report.

## Conflicts of Interest

The authors declare no conflicts of interest.

## Supporting information


**Table S1:** PCR and RT‐qPCR primers used in this study.
**Table S2:** Cryo‐substitution and resin‐embedding protocol after high pressure freezing.
**Figure S1:** Generation and characterization of *AGS* promoter‐*eGFP* strains. (A) The *eGFP* gene was driven by the promoters of the α‐1,3‐glucan synthase genes *AGS1*, *AGS2*, or *AGS3*. T*nos* served as the terminator. The nourseothricin‐resistance cassette was used to select transformants. The *Dra*I, *Hind*III, and *Bsp*TI restriction sites in P_*AGS1*_:*eGFP*, P_*AGS2*_:*eGFP*, and P_*AGS3*_:*eGFP* constructs are indicated. The black line under the nourseothricin‐resistance cassette represents the binding site of the probe used in Southern blots. (B) Genomic Southern blots of P_*AGS1*_:*eGFP*, P_*AGS2*_:*eGFP*, and P_*AGS3*_:*eGFP* strains. The restriction endo‐nucleases used are indicated. Transformants harboring single constructs are indicated by an asterisk. (C) Phenotype of the WT and a P_*AGS1*_:*eGFP*, P_*AGS2*_:*eGFP*, and a P_*AGS3*_:*eGFP* strain growing on OMA. (D) Daily growth rates of the WT and the P_*AGS1*_:*eGFP*, P_*AGS2*_:*eGFP*, and P_*AGS3*_:*eGFP* strains on OMA (plain bars) and PDA (hatched bars). (E) Number of conidia formed by the WT and the P_*AGS1*_:*eGFP*, P_*AGS2*_:*eGFP*, and P_*AGS3*_:*eGFP* strains on OMA after 12 days. Error bars in (D, E) indicate standard deviations. (F) Anthracnose disease symptoms caused by the WT and P_*AGS1*_:*eGFP*, P_*AGS2*_:*eGFP*, and P_*AGS3*_:*eGFP* strains on non‐wounded and wounded leaves of *Zea mays* cv. Mikado plants. A mock‐inoculated leaf segment served as control. Leaves were photographed at 4 dpi.
**Figure S2:** Generation of single, double, and triple deletion mutants lacking α‐1,3‐glucan synthase genes *AGS1*, *AGS2*, and/or *AGS3* by homologous recombination. Generation of Δ*ags1*, Δ*ags2* and Δ*ags3* strains are shown in (A–F). The Δ*ags1* mutant served as the recipient for the *AGS2* (G, H) and *AGS3* (I, K) deletion constructs, yielding the Δ*ags1,2* and Δ*ags1,3* mutants, respectively, and the Δ*ags2* mutant was the recipient for the *AGS3* deletion construct (L, M), yielding the Δ*ags2,3* mutant. The Δ*ags1,2,3* triple mutant was generated by transforming the *AGS3* deletion construct into Δ*ags1,2* cells. Genomic Southern blots (B, D, F, H, K, M, and O) confirmed site‐directed integration of constructs. The black lines in (A, C, and E) represent the binding sites of the probes used in Southern blots shown in (B, D, and F), respectively.
**Figure S3:** Germinating conidia of the *C. graminicola* WT strain and mutants lacking *AGS1*, *AGS2*, *AGS3*, or all three *AGS* genes. (A) Micrographs of a conidium of the WT and the Δ*ags3* strain. co, conidium; gt, germ tube. Arrows point towards germ tubes emerging from the conidium of the Δ*ags3* mutant. Size bars represent 10 μm. (B) Quantification of the fraction of conidia producing two or more germ tubes is shown. One‐hundred conidia of each strain were counted, and the experiment was repeated three times (biological replicates). Error bars represent standard deviations, asterisks indicate statistical significance at *p* < 0.05.
**Figure S4:** Effect of the β‐1,3‐glucan synthesis inhibitor Caspofungin and of the chitin synthesis inhibitor Nikkomycin Z on colony growth and cell wall properties of the WT strain and α‐1,3‐glucan‐deficient mutants. As compared with growth on PDA, colony diameters of WT and mutants were strongly reduced by Caspofungin (0.032 μg/mL). On PDA, the WT developed smooth hyphae with intact hyphal tips (black arrowheads), whereas the mutant formed small pigmented cells (dark arrows) and slightly swollen hyphal tips (white arrows). In the presence of Caspofungin, the WT strain formed apparently intact (black arrowheads) hyphal tips, whereas intact (black arrowhead) as well lysed hyphal tips (white arrowhead) were observed in the Δ*ags1,2,3* strain. In comparison, Nikkomycin Z (24.7 μg/mL) marginally affected colony development of the WT strain, but strongly inhibited growth of the Δ*ags1,2,3* mutant. Ballooning was observed in both WT and mutant (white arrows), and strong pigmentation (black arrows) occurred. In the presence of Nikkomycin Z, the Δ*ags1,2,3* mutant formed setae (thin white arrow) and large numbers of conidia (thin black arrow). Size bars represent 10 μm.
**Figure S5:** FRAP experiment (A) Fluorescence image before photobleaching, and after photobleaching and fluorescence recovery. The white box marks the initially bleached region. Size bars represent 10 μm. (B) Exemplary fluorescence intensity time‐trajectory (red symbols) and the fitting curve (black line) for determination of the (slow) mobile fraction. The time‐point of bleaching and the mobile fraction are indicated. (C) Molecular mass‐dependence of the mean hydrodynamic radius for FITC‐dextrans as provided by the manufacturer.

## Data Availability

The data that support the findings of this study are available on request from the corresponding author. The data are not publicly available due to privacy or ethical restrictions.
