# Supplementary material for: α‐1,3‐Glucan Is Required for Cell Wall Integrity and Full Virulence of Colletotrichum graminicola, but Not for Evasion of PAMP‐Triggered Immunity
Source: Mol Microbiol. 2026 Sep 10;126(5):482–502. doi: 10.1111/mmi.70114 (PMC13725317; doi:10.1111/mmi.70114)
Supplement: Supplementary file 1 [file MMI-126-482-s001.docx]

**Supplemental Material**

**α-1,3-glucan is required for cell wall integrity and full virulence of *Colletotrichum graminicola*, but not for evasion of PAMP-triggered immunity**

Iris Gase^1^, Lala Aliyeva-Schnorr^1^, Michael Melzer^2^, Maria Ott^3^, Julia Haufe^1^, Matthias Menzel^4^, Timo Engelsdorf^5^, Christiane Rohrbach^5^, Alan de Oliveira Silva^1^, Lars M. Voll^5^, Alexander Mickel^1^, Maximilian Groß^1^, Mamoru Wakayama^6^, and Holger B. Deising^1,*^

^1^ Martin-Luther-University Halle-Wittenberg, Faculty of Natural Sciences III, Institute for Agricultural and Nutritional Sciences, Chair for Phytopathology and Plant Protection, Betty-Heiman-Str. 3, D-06120 Halle (Saale), Germany

^2^ Leibniz Institute of Plant Genetics and Crop Plant Research (IPK) OT Gatersleben, Corrensstraße 3, D-06466 Seeland, Germany

^3^ Martin-Luther-University Halle-Wittenberg, Faculty of Natural Sciences I, Institute for Biochemistry and Biotechnology, Kurt-Mothes-Str. 3; D-06112 Halle (Saale); Germany

^4^ [Fraunhofer Institute for Microstructure of Materials and Systems IMWS](https://www.imws.fraunhofer.de/en.html); Walter-Hülse-Str. 1; D-06112 Halle (Saale); Germany

^5^ Philipps-University Marburg, Department of Biology, Chair for Molecular Plant Physiology, Karl-von-Frisch-Str. 8, D-35032 Marburg, Germany

^6^ Ritsumeikan University, Faculty of Life Sciences, Department of Biotechnology, Kusatsu, Shiga 525-8577, Japan

**Supplemental Table S1: PCR and RT-qPCR primers used in this study**

| ***AGS1* primers** | **sequences (5`→ 3`)** |
| --- | --- |
| AGS1-P-fw | CAAGTACATGGGCATGTAACTC |
| AGS1-P-fw.nest | TCAACGTGACGGGCAATCTC |
| AGS1-P-hyg.rev | GTGCAACTGACAGTCGTACACAGAGCACGAGCAAATACAG |
| AGS1-T-hyg.fw | TCTGGAGTCTCACTAGCTTCTGCGGAGAGGAGGTTTAAGC |
| AGS1-T-rev | AACAGGCCGCATATGAC |
| AGS1-T-rev.nest | ACCTCGCGTGCTATAGATTC |
| AGS1-Sonde-fw | CAGTTTCTCGGGTTTGATGG |
| AGS1-Sonde-rev | GACCAGTATTGCACCATGAC |

| ***AGS2* primers** | **sequences (5`→ 3`)** |
| --- | --- |
| AGS2-P-fw | TCTTGTTGGCCAGAGTCCAC |
| AGS2-P-fw.nest | CTGAACCTGGCCTATAGC |
| AGS2-P-nours.rev | GTGCAACTGACAGTCGTACACATGAACTGGCGGAAGC |
| AGS2-T-nours.fw | GTCTGGAGTCTCACTAGCTTGGAGGATAGAGCATTTCCTG |
| AGS2-T-rev | AAGCTAGAGGGGACCAAATC |
| AGS2-T-rev.nest | CACCAGCAGTTCTTGTACTTG |
| AGS2-Sonde-fw | CGGTACTGGAGGTACTTCTTG |
| AGS2-Sonde-rev | ACGGTAAGAAGGGTGTCAAG |

| ***AGS3* primers** | **sequences (5`→ 3`)** |
| --- | --- |
| AGS3-P-fw | CTCCAACGTCTCGTATTCTC |
| AGS3-P-fw.nest | CCACCGTATTGGTCAGTC |
| AGS3-P-G418.rev | GTGCAACTGACAGTCGTACAGACGAAGTGAAGCTCCAATG |
| AGS3-T-G418.fw | GTCTGGAGTCTCACTAGCTTAGGCGCCATGCAAGAATGTG |
| AGS3-T-rev | CGGTGGGAACGGCTTCTATG |
| AGS3-T-rev.nest | GCTCGAATCAAGCCAGTC |
| AGS3-Sonde-fw | CGTCGATCCGTAATTGGATG |
| AGS3-Sonde-rev | CGCAATGTCCATCCATTTCG |

**Primers used to amplify**

***AGS* promoter regions	sequences (5`→ 3`)**

PAGS1_Fw	ACGTCATGGTGCAATACTGGT

PAGS1_Rv	TCCTCCCTGGCTATCCGTA

PAGS2_Fw	GCCGTCAACATTACGAAGCC

PAGS2_Rv	CAATGGGGAAACGGTTGCAG

PAGS3_Fw	GCTGTCACTCACTGTTTTCTTGT

PAGS3_Rv	AGTTGTCGCCTGCATAGCAT

| **General primers** | **sequences (5`→ 3`)** |
| --- | --- |
| G418-probe-Fw | GGATTGCACGCAGGTTCTCC |
| G418-probe-Rv | GGAGCGGCGATACCGTAAAG |
| Hyg-probe-Fw | TGAACTCACCGCGACGTCTG |
| Hyg-probe-Rv | TCGGCCCAAAGCATCAGCTC |
| Nours-probe-Fw | CTCTTGACGACACGGCTTAC |
| Nours-probe-Rv | GGCAGGGCATGCTCATGTAG |
| uni-G418.F1 | TGTACGACTGTCAGTTGCACAGCGCGTTGTTGGATTAAG |
| uni-G418.R1 | AAGCTAGTGAGACTCCAGACCTCAGAAGAACTCGTCAAGAAG |
| uni-hyg.F1 | TGTACGACTGTCAGTTGCACTGACCGGTGCCTGGATCTTC |
| uni-hyg.R1 | AAGCTAGTGAGACTCCAGACGGTCGGCATCTACTCTATTCC |
| uni-nours.F1 | TGTACGACTGTCAGTTGCACATTCGGGCCGGATTG |
| uni-nours.R1 | AAGCTAGTGAGACTCCAGACACCGATGAAACGATTCTCAAC |

**RT-qPCR primers	sequences (5`→ 3`)**

ZmCTB_RT-Fw	ACGACCTGCCGACGTTCTTC

ZmCTB_RT-Rv	CGGCTCCAAGTCGTAGAATG

ZmPOX365_RT-Fw	GAGATGACGACCGCTCCCATTG

ZmPOX365_RT-Rv	AGCGGGCTTATGTTGCCCATC

ZmPOX346_RT-Fw	TTCCTGATGCCACCAAGGGTTC

ZmPOX346_RT-Rv	GAGGGCAACGATGTCCTGATCAC

ZmCS631_RT-Fw	CATCCTGCTGGCCTCGATCTTCTC

ZmCS631_RT-Rv	CAGTTGCAGTCCAGGCCACACTC

ZmChit633_RT-Fw	GTCACCGGCTCCTTCTTCAAC

ZmChit633_RT-Rv	GCTCCGGGTGTAGAAGTTCTTG

ZmBGlu837_RT-Fw	CTTCCCGGCGAAGCCGATG

ZmBGlu837_RT-Fw	CAACTACAAGTACGCGCCGTTC

ZmTPS2_RT-Fw	GTCCGTACTTTGGCGGCATCATC

ZmTPS2_RT-Rv	TCCGTTTGACGTCCGTCGTCAG

ZmTPS7_RT-Fw	TGTACAATGCGGCTCACATGGC

ZmTPS7_RT-Rv	CTGACCTCGCCTTTGCTGATG-3’

ZmGLP512_RT-Fw	AGGCCATCGCCATGACGCTCTTC

ZmGLP512_RT-Rv	TGGGTGCGAAGTTTGCCTTGATG

**Supplemental Table S2:** **Cryo-substitution and resin-embedding protocol after high pressure freezing.**

| Cryo Substitution Reagent | Temperature  [°C] | Time  [h] | Notes |
| --- | --- | --- | --- |
| 100 % acetone | -80 | 96 * |  |
| 100 % acetone | -80 to -70 | 6 |  |
| 100 % acetone | -70 | 24 |  |
| 100 % acetone | -70 to -50 | 6 |  |
| 100 % acetone | -50 | 24 |  |
| 100 % acetone | -50 to -35 | 6 |  |
| 100 % acetone | -35 | 12 |  |
| 100 % acetone | -35 to -10 | 6 |  |
| 100 % acetone | -10 to 20 | 2 |  |
| 100 % acetone | 20 | 0.5 |  |
| 25% of HM20 resin in 100% acetone | 20 | over night | on shaker |
| 50% of HM20 resin in 100 % acetone | 20 | 3 | on shaker |
| 75% of HM20 in 100 % acetone | 20 | 3 | on shaker |
| 100% of HM20 resin | 20 | o.n. | on shaker |
| UV-polymerization of samples in gelatin capsules for 72 hrs at -10 °C in a Leica automated freeze substitution unit (AFS) | | | |

* To optimise the subsequent freezing substitution process, the samples were removed from the aluminium plates after 24 hours.

**Supplemental Figures S1 to S5**

**Supporting Fig. S1.** Generation and characterization of *AGS* promoter-*eGFP* strains. **A.** The *eGFP* gene was driven by the promoters of the α-1,3-glucan synthase genes *AGS1*, *AGS2*, or *AGS3*. T*nos* served as the terminator. The nourseothricin-resistance cassette was used to select transformants. The *Dra*I, *Hind*III, and *Bsp*TI restriction sites in P*_AGS1_*:*eGFP*, P*_AGS2_*:*eGFP*, and P*_AGS3_*:*eGFP* constructs are indicated. The black line under the nourseothricin-resistance cassette represents the binding site of the probe used in Southern blots. **B.** Genomic Southern blots of P*_AGS1_*:*eGFP*, P*_AGS2_*:*eGFP*, and P*_AGS3_*:*eGFP* strains. The restriction endo-nucleases used are indicated. Transformants harboring single constructs are indicated by an asterisk. **C.** Phenotype of the WT and a P*_AGS1_*:*eGFP*, P*_AGS2_*:*eGFP*, and a P*_AGS3_*:*eGFP* strain growing on OMA. **D.** Daily growth rates of the WT and the P*_AGS1_*:*eGFP*, P*_AGS2_*:*eGFP*, and P*_AGS3_*:*eGFP* strains on OMA (plain bars) and PDA (hatched bars). **E.** Number of conidia formed by the WT and the P*_AGS1_*:*eGFP*, P*_AGS2_*:*eGFP*, and P*_AGS3_*:*eGFP* strains on OMA after 12 days. Error bars in (**D**) and (**E**) indicate standard deviations. **F.** Anthracnose disease symptoms caused by the WT and P*_AGS1_*:*eGFP*, P*_AGS2_*:*eGFP*, and P*_AGS3_*:*eGFP* strains on non-wounded and wounded leaves of *Zea mays* cv. Mikado plants. A mock-inoculated leaf segment served as control. Leaves were photographed at 4 dpi.

**Supporting Fig. S2.** Generation of single, double, and triple deletion mutants lacking α-1,3-glucan synthase genes *AGS1*, *AGS2*, and/or *AGS3* by homologous recombination. Generation of Δ*ags1*, Δ*ags2* and Δ*ags3* strains are shown in **A** to **F**. The Δ*ags1* mutant served as the recipient for the *AGS2* (**G**, **H**) and *AGS3* (**I**, **K)** deletion constructs, yielding the Δ*ags1,2* and Δ*ags1,3* mutants, respectively, and the Δ*ags2* mutant was the recipient for the *AGS3* deletion construct (**L**, **M**), yielding the Δ*ags2,3* mutant. The Δ*ags1,2,3* triple mutant was generated by transforming the *AGS3* deletion construct into Δ*ags1,2* cells. Genomic Southern blots (B, D, F, H, K, M, and O) confirmed site-directed integration of constructs. The black lines in A, C and E represent the binding sites of the probes used in Southern blots shown in B, D and F, respectively.

**Supporting Fig. S3.** Germinating conidia of the *C. graminicola* WT strain and mutants lacking *AGS1*, *AGS2*, *AGS3*, or all three *AGS* genes. **A.** Micrographs of a conidium of the WT and the Δ*ags3* strain. co, conidium; gt, germ tube. Arrows point towards germ tubes emerging from the conidium of the Δ*ags3* mutant. Size bars represent 10 µm. **B.** Quantification of the fraction of conidia producing two or more germ tubes is shown. One-hundred conidia of each strain were counted, and the experiment was repeated three times (biological replicates). Error bars represent standard deviations, asterisks indicate statistical significance at *P* < 0.05.

**Supporting Fig. S4.** Effect of the β-1,3-glucan synthesis inhibitor Caspofungin and of the chitin synthesis inhibitor Nikkomycin Z on colony growth and cell wall properties of the WT strain and α-1,3-glucan-deficient mutants. As compared with growth on PDA, colony diameters of WT and mutants were strongly reduced by Caspofungin (0.032 µg/ml). On PDA, the WT developed smooth hyphae with intact hyphal tips (black arrowheads), whereas the mutant formed small pigmented cells (dark arrows) and slightly swollen hyphal tips (white arrows). In the presence of Caspofungin, the WT strain formed apparently intact (black arrowheads) hyphal tips, whereas intact (black arrowhead) as well lysed hyphal tips (white arrowhead) were observed in the Δ*ags1,2,3* strain. In comparison, Nikkomycin Z (24.7 µg/ml) marginally affected colony development of the WT strain, but strongly inhibited growth of the Δ*ags1,2,3* mutant. Ballooning was observed in both WT and mutant (white arrows), and strong pigmentation (black arrows) occurred. In the presence of Nikkomycin Z, the Δ*ags1,2,3* mutant formed setae (thin white arrow) and large numbers of conidia (thin black arrow). Size bars represent 10 µm.

**Supporting Fig. S5.** FRAP experiment **A.** Fluorescence image before photobleaching, and after photobleaching and fluorescence recovery. The white box marks the initially bleached region. Size bars represent 10 µm. **B.** Exemplary fluorescence intensity time-trajectory (red symbols) and the fitting curve (black line) for determination of the (slow) mobile fraction. The time-point of bleaching and the mobile fraction are indicated. **C.** Molecular mass-dependence of the mean hydrodynamic radius for FITC-dextrans as provided by the manufacturer.
